# Crystalline‐Amorphous Heterostructure: A Novel Configuration for Silver Nanoclusters

**DOI:** 10.1002/advs.202501186

**Published:** 2025-06-29

**Authors:** Jia‐Hong Huang, Yao Cui, Peng Luo, Zhao‐Yang Wang, Shuang‐Quan Zang

**Affiliations:** ^1^ College of Chemistry Zhengzhou University Zhengzhou 450001 China; ^2^ College of Chemistry and Chemical Engineering Henan Polytechnic University Jiaozuo 454000 China

**Keywords:** assembly, heterostructure, metal nanocluster, silver nanocluster

## Abstract

Heterostructured composite materials with multiple components have potential applications in diverse aspects, as they tend to exhibit superior physicochemical properties than the sum of their single counterparts. However, the fabrication of heterostructures composed of atomic‐precise metal nanoclusters (NCs) is seldom reported owing to their inherent instability, leading to the decomposition of metal NCs during the formation of peripheral layers. Here, a spontaneously formed amorphous‐crystalline heterostructured **Ag_40_@Ag_12_** is discovered, wherein the amorphous Ag_40_ NCs are enveloped by a single crystal of Ag_12_ NCs. The formation of **Ag_40_@Ag_12_** involves three stages, including initial crystallization of Ag_40_ NCs, partial decomposition (self‐sacrifice) of Ag_40_ NCs enabling Ag_12_ assembly, and epitaxial‐growth crystallization of the Ag_12_ NCs. The partially decomposed Ag_40_ NCs provide starting materials for the formation and crystallization of Ag_12_ NCs to encapsulate the Ag_40_ seeds, while the residual **Ag_40_** loses its crystallinity in solution simultaneously. This work not only reports a novel class of heterostructured materials for the first time but also provides new insights into the macroscopic co‐assembly of distinct silver clusters.

## Graphical abstract

This study presents a spontaneously formed amorphous‐crystalline heterostructure composite composed of atomically precise carboranylthiolate‐protected silver NCs, demonstrating not only a unique co‐assembly of distinct metal NCs at the macroscopic level but also promising potential applications of metal cluster‐based materials.

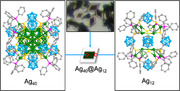

## 1 Introduction

Atomically precise metal nanoclusters (NCs) are associated with intriguing physicochemical properties and discrete electronic energy levels originating from the quantum size effect, and thus the investigations of the structure–property correlations at the atomic level attract great attention.^[^ ^1^, ^2^, ^3^, ^4^, ^5^, ^6^, ^7^, ^8^ ^]^ In addition to the discrete NC, the NC‐assembled materials with newly generated properties through collective behavior have emerged as research hotspots recently.^[^ ^9^, ^10^, ^11^, ^12^, ^13^, ^14^, ^15^, ^16^, ^17^, ^18^, ^19^ ^]^ For instance, Zhao′s group constructed the silver cluster aggregates with helical nanofibers morphology, which exhibits superior circularly polarized luminescence performance.^[^ ^20^ ^]^ Yam′s group exploited chiral anions to tune the morphology of the Au‐NC nanocrystals from rhombic to strip and quasi‐hexagonal nanosheets.^[^ ^21^ ^]^ Tang reported the self‐assembly of chiral gold clusters into crystalline nanocubes recording the highest absorption anisotropy factor (7 × 10^−3^) among the coinage‐metal clusters.^[^ ^22^ ^]^ Besides weak intercluster interactions, our group first proposed the covalent assembly of silver NCs and reported a series of porous cluster‐assembled materials, which broke the common dense packing of metal NCs and brought out new properties.^[^ ^23^, ^24^, ^25^, ^26^ ^]^ Despite the progress, new assembly forms are still expected to endow silver NCs with intriguing properties.^[^ ^27^, ^28^, ^29^, ^30^, ^31^, ^32^, ^33^, ^34^, ^35^ ^]^

Heterostructure composites are regarded as an important strategy to impart multifunctionality to crystalline materials.^[^ ^36^ ^]^ Extensive efforts, especially for the epitaxial growth methods, have been devoted to the rational design and modulation of the components of the crystalline‐material composite.^[^ ^37^, ^38^, ^39^ ^]^ However, the heterostructured composite crystals based on metal NCs have not yet been achieved due to their poor stability, which often drives the decomposition or NC‐to‐NC transformations during the fabrication of the peripheral layers.

Herein, a spontaneous formation of an amorphous‐crystalline heterostructured **Ag_40_@Ag_12_** was discovered, wherein the carboranylthiolate‐protected Ag_12_ NCs in the single‐crystal phase envelop the amorphous Ag_40_ NCs. The sharp distinction in phases was verified through a cryo‐transmission electron microscope (cryo‐TEM). Upon modification of the synthetic procedure, the composite silver NCs could be isolated in single crystals of **Ag_12_** and **Ag_40_**. The Ag_12_ NC adopts a nonspherical structure containing Ag(I)–thiolate–phosphine motifs, while the superatomic Ag_40_ NC features a multilayer core–shell structure composed of a Platonic icosahedron Ag_12_, a Platonic dodecahedron Ag_20_ shell, and a Platonic hexahedra Ag_8_ shell from inside out, which is reported for the first time. Further investigation elucidates a process of Ag_40_ crystallization→Ag_40_ partial decomposition and Ag_12_ formation→Ag_12_ recrystallization in the formation of the heterostructured **Ag_40_@Ag_12_**. This work first fabricated the atomically precise metal NC‐based heterostructured material, which is expected to not only provide a new type of metal cluster‐assembled materials but also broaden the potential applications of metal NCs.

## 2 Results and Discussion

### 2.1 Synthesis and Characterization

**Scheme 1:**
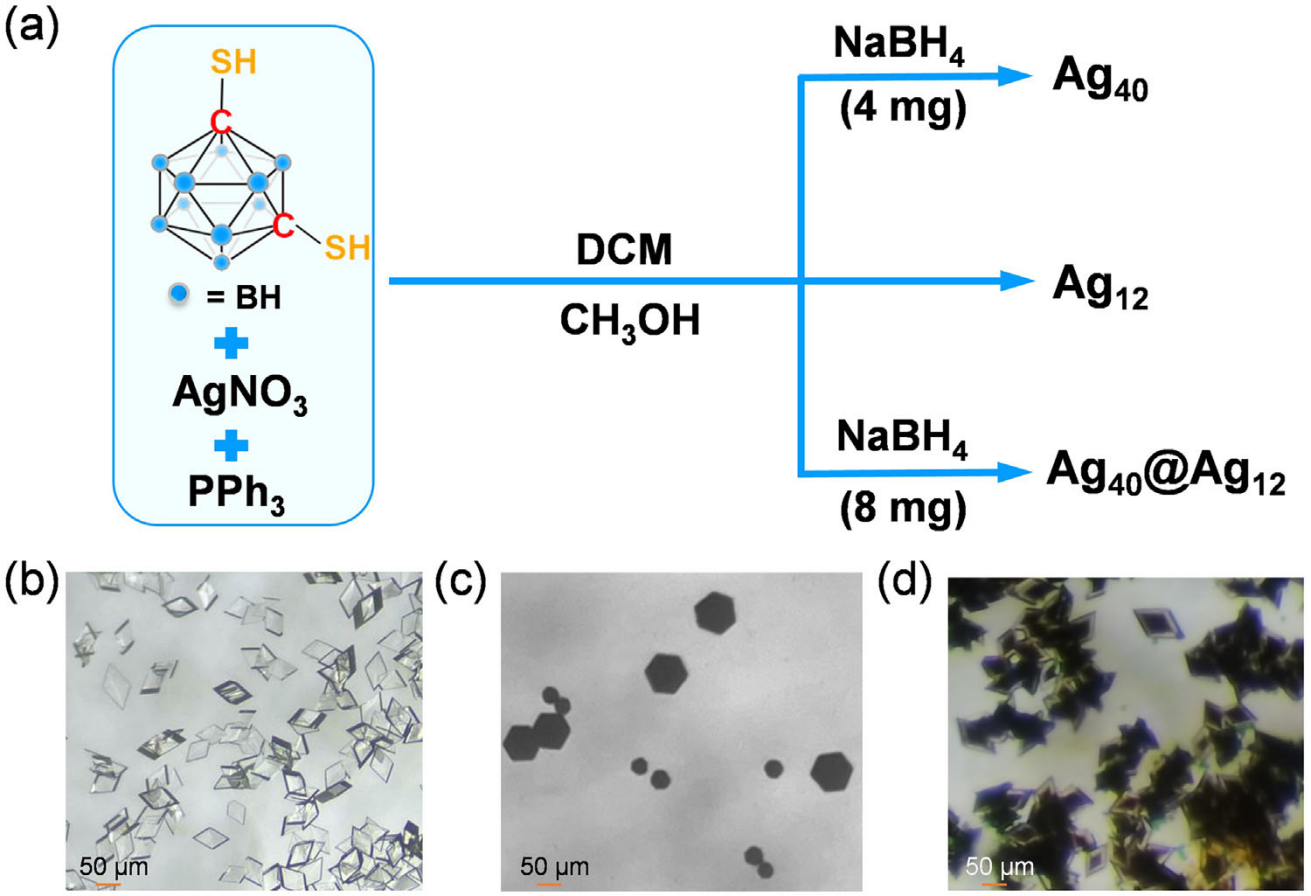
a) The synthetic route of Ag_40_, Ag_12_, and Ag_40_@Ag_12_. b–d) Images of the Ag_12_ (b), Ag_40_ (c), and Ag_40_@Ag_12_ (d) crystals. Color labels: blue, BH vertex of carborane.

The synthetic details of 1,7‐bis(mercapto)‐*m*‐carborane (C_2_B_10_H_12_S_2_), **Ag_12_**, **Ag_40_**, and **Ag_40_@Ag_12_** are described in the Supporting Information (Section S2 and Figures S1–S3, Supporting Information). In brief, for the synthesis of **Ag_12_**, silver nitrate, C_2_B_10_H_12_S_2_, and triphenylphosphine (PPh_3_) were successively added in a mixed solvent of methanol‐dichloromethane to afford the Ag−S−P complex (**Scheme** **1**). Colorless rhombic crystals of **Ag_12_** were obtained after 2 days of evaporation (Scheme 1b; Figure S4, Supporting Information). The synthesis of **Ag_40_** involved the chemical reduction of the Ag‐S‐P complex in DMF by an aqueous NaBH_4_ (0.1 mmol), and a mixture of black hexagonal **Ag_40_** and rhombic **Ag_12_** crystals were grown by slow evaporation of the solution (Figure S5, Supporting Information). Pure crystals of **Ag_40_** could also be obtained by diffusing diethyl ether into the DMF solution (Scheme 1c; Figure S6, Supporting Information). The synthetic method of **Ag_40_@Ag_12_** was similar to that of **Ag_40_** except for the increased amount of NaBH_4_ to 0.2 mmol, and the eye‐catching rhombic crystals with black core and colorless shell were obtained (Scheme 1d; Figure S7, Supporting Information). Such heterostructure assembly of distinctive silver NCs has been scarcely reported. Energy dispersive spectrometry (EDS) and X‐ray photoelectron spectroscopy (XPS) identified that **Ag_12_, Ag_40_, and Ag_40_@Ag_12_** include Ag, S, P, B, and C atoms (Figures S8–S10, Supporting Information). Powder X‐ray diffraction (PXRD) patterns confirmed the phase purity of **Ag_12_** and **Ag_40_** (Figure S11, Supporting Information). Electrospray ionization mass (ESI‐MS) spectra proved that the Ag_40_ NC was anionic (−3 charged state) with 18 valence electrons (Figure S12, Supporting Information). Ag_40_ nanoclusters dispersed in solution undergo PPh_3_ dissociation, which renders them sensitive to external stimuli (oxygen and solutions) and prone to decomposition (Figure S13, Supporting Information). By comparison, Ag_40_ nanocrystals are more stable under ambient conditions because the PPh_3_ will not dissociate in the solid state, and the aggregation‐induced barrier to oxygen may also account for its enhanced stability.^[^ ^40^ ^]^ As shown in Figure S14 (Supporting Information), Ag_40_ nanocrystals retained the absorption characteristic after one month. The thermogravimetric analysis (TGA) data indicate that the Ag_40_ nanocrystals are thermally stable below 205 °C (Figure S15, Supporting Information).

**Figure 1:**
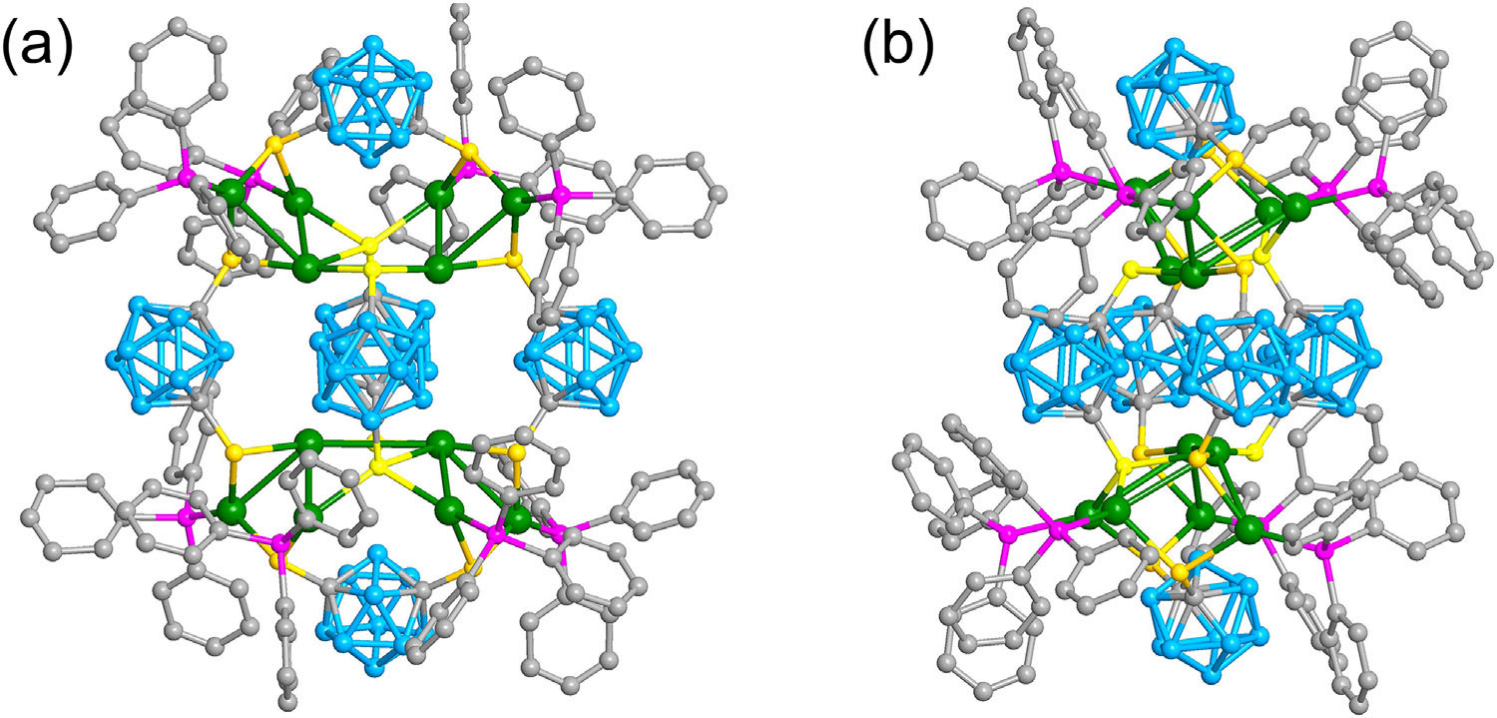
a) The overall structure of Ag_12_. b) Side view of Ag_12_. Color labels: green, Ag; yellow, S; purple, P; grey, C; cyan, B. All hydrogen atoms are omitted for clarity.

Single‐crystal structure analyses revealed that **Ag_12_** crystallized in the triclinic space group *P* $\bar{1}$ and the asymmetric unit contains a complete cluster with a formula of Ag_12_(C_2_B_10_H_10_S_2_)_6_(PPh_3_)_4_ (**Figure** **1**a).^[^ ^41^ ^]^ No counter ion was found in the lattice implying that the Ag_12_ NC is electroneutral. Each Ag_12_ NC contained 12 silver atoms co‐protected by 12 thiolates and 8 phosphines, which could be viewed as two centrosymmetric Ag_4_C_2_B_10_H_10_S_2_(PPh_3_)_2_ units (Figure S16a, Supporting Information) connected by two C_2_B_10_H_10_S_2_ ligands, resulting in an Ag_8_(C_2_B_10_H_10_S_2_)_4_(PPh_3_)_4_ metallacycle (Figure S16b, Supporting Information). The metallacycle was consolidated by two Ag_2_C_2_B_10_H_10_S_2_ complexes from the top and bottom, respectively (Figure S16c, Supporting Information). Each thiolate connected two adjacent silver atoms while the phosphines only ligated to one silver atom. Ag‐Ag interactions exist between Ag_4_C_2_B_10_H_10_S_2_(PPh_3_)_2_ and Ag_2_C_2_B_10_H_10_S_2_ with separations in the range of 3.09–3.17 Å.

**Figure 2:**
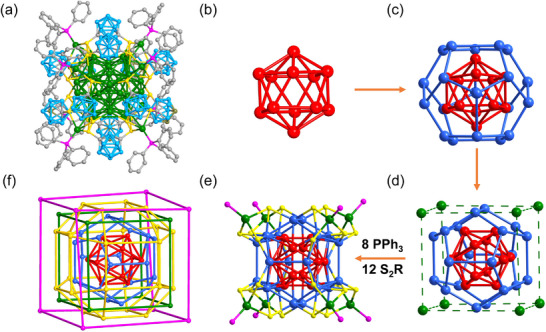
a) The overall crystal structure of Ag_40_. b) The hollow icosahedral Ag_12_ core. c) The interpenetrating dodecahedral Ag_20_ and icosahedral Ag_13_ form a core–shell structure. d) The hexahedral Ag_12_@Ag_20_@Ag_8_ metal framework. e) The coordination modes of thiolate and phosphine ligands. f) Schematic illustration of the Ag_12_@Ag_20_@Ag_8_@S_24_@P_8_. Color labels: red, blue, and green, Ag; yellow, S; purple, P; grey, C; cyan, B. All hydrogen atoms are omitted for clarity.

As revealed by X‐ray crystallography, **Ag_40_** crystallized in the trigonal space group *P* $\bar{3}$, and contains an anionic cluster with a formula of [Ag_40_(C_2_B_10_H_10_S_2_)_12_(PPh_3_)_8_]^2^^−^ accompanied by two Ag(PPh_3_)_4_^+^ counterions (Figure S17, Supporting Information). The Ag_40_ anion could be regarded as an Ag_12_@Ag_20_@Ag_8_ multilayer core–shell architecture co‐protected by twelve dithiolate and eight phosphine ligands (**Figure** **2**a). Specifically, the innermost hollow Ag_12_ icosahedron is encapsulated by the Ag_20_ dodecahedron (icosahedron@ dodecahedron) (Figure 2b,c). Each Ag atom from the inner Ag_12_ icosahedron points to the center of the dodecahedron faces. The Ag···Ag distances in the Ag_12_ icosahedron hold an average value of 2.838 Å, which is shorter than those of 2.880 Å in bulk silver. The average Ag···Ag distance in the Ag_20_ dodecahedron was 2.942 Å. The *I_h_* symmetric Ag_12_@Ag_20_ kernel was surrounded by eight Ag atoms, which formed a cube with an average Ag···Ag distance of 8.515 Å (Figure 2d). Six edges of the Ag_20_ dodecahedron are located in the corresponding faces of the cube. Each *µ*_3_‐S in the ligand connected two adjacent Ag atoms from the Ag_20_ dodecahedron and one Ag atom from the Ag_8_ cube (Figure 2e; Figure S18, Supporting Information). The arrangement of the 24 S atoms exhibits a rhombicuboctahedral polyhedron (Figure 2f). In addition, the eight PPh_3_ ligands anchor to the corners of the cube (Figure 2e,f). Notably, metal clusters composed of icosahedron@dodecahedron or dodecahedron@cube geometries have been previously reported.^[^ ^42^, ^43^, ^44^, ^45^, ^46^, ^47^, ^48^ ^]^ However, their combination was first observed in this study, although such a metal framework of Ag_40_ has been predicted in our previous review.^[^ ^49^ ^]^ Moreover, the similar bidentate ligand 9,12‐dimercapto‐*o*‐carborane afforded the Ag_33_(C_2_B_10_H_10_S_2_)_12_ NC with an icosahedron@dodecahedron structure,^[^ ^50^ ^]^ suggesting that the distance between the two thiols makes a great impact on the geometry of metal NC.

**Figure 3:**
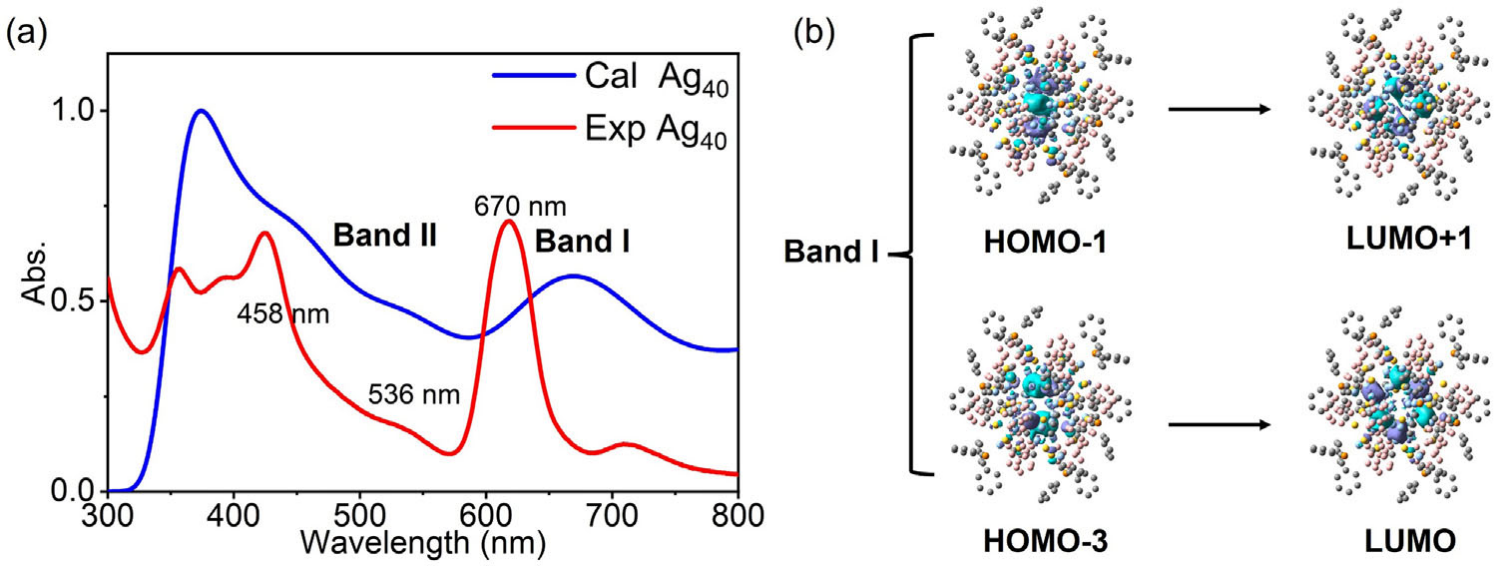
a) Experimental and calculated absorption spectra of **Ag_40_**. b) Transition‐involved molecular orbitals of **Ag_40_**.

According to the spherical jellium model, the Ag_40_ NC possesses 18 free valence electrons, and the electronic configuration corresponds to 1S^2^1P^6^1D^10^, which is also confirmed by density functional theory (DFT) calculations. Compared to the original crystal structure, DFT‐optimized Ag_40_ NC maintains its *S*_6_ symmetry. The HOMO−4 to HOMO of the Ag_40_ NC exhibit the characteristic of 1D superatomic orbitals, while LUMO+3 corresponds to the 2S orbital, and the LUMO to LUMO+2, and LUMO+4 to LUMO+7 are 1F superatomic orbitals (Figure S19, Supporting Information). The five 1D orbitals are separated into two groups: the triply degenerated HOMO−2 to HOMO, and the doubly degenerated HOMO‐3 and HOMO‐4. Time‐dependent density functional theory (TD‐DFT) simulated absorption spectrum displays four distinct peaks at 374, 458, 536, and 670 nm (**Figure** **3**a). The band I at ≈670 nm in the simulated spectrum corresponds to the experimental absorption at 618 nm, which mainly involves the HOMO‐1 to LUMO+1 and HOMO‐3 to LUMO transitions (Figure 3b). These electronic transitions are typically attributed to the charge transfer within the metal kernel. The shoulder peaks (band II) at 458 and 536 nm mainly involve the ligand‐to‐metal charge transfer.

### 2.2 Characterization of the Ag_40_@Ag_12_ Heterostructure

To our surprise, the SCXRD revealed a good single‐crystallinity of the heterostructured Ag_40_@Ag_12_, which crystallized in the triclinic space group *P* $\bar{1}$. The structure determination only verifies the existence of Ag_12_ NCs in the lattice, which are almost the same as those in Ag_12_ except for the slight changes in bond lengths and angles. The cell volume of Ag_40_@Ag_12_ was less than that of Ag_12_, while the density represented an opposite trend. Viewing from the *z*‐axis of Ag_12_ and Ag_40_@Ag_12_, the Ag_12_ NCs are packed into hexagonal patterns and each of them is surrounded by six Ag_12_ NCs in the same layer (Figure S20, Supporting Information). For Ag_40_@Ag_12_, weak intermolecular forces including C−H···*π* and B‐H···*π* were found between the Ag_12_ clusters, and the void between the clusters was not suitable for accommodating additional metal clusters. These findings suggest that the Ag_40_ NCs in Ag_40_@Ag_12_ are amorphous.

**Figure 4:**
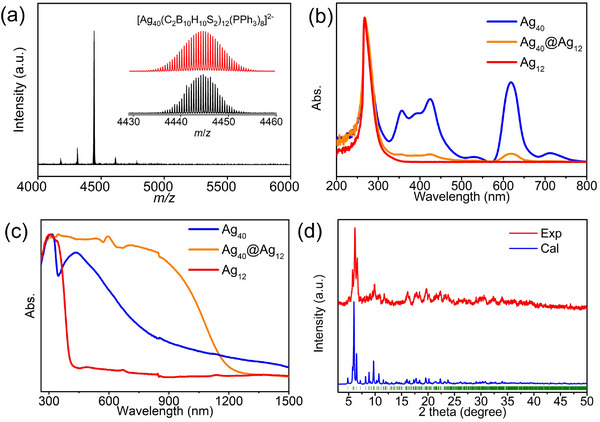
a) Negative‐mode ESI‐MS spectrum of Ag_40_@Ag_12_. Inset: The enlarged portion of the spectrum showed the measured (black) and simulated (red) isotopic distribution patterns. b) UV–vis spectra of Ag_12_, Ag_40_, and Ag_40_@Ag_12_. c) UV–vis diffuse reflectance spectra of **Ag_12_**, **Ag_40_**, and **Ag_40_@Ag_12_** crystals. d) PXRD pattern of **Ag_40_@Ag_12_** crystals.

To further identify the constituents of **Ag_40_@Ag_12_**, ESI‐MS was performed. ESI‐MS spectrum of **Ag_40_@Ag_12_** showed a predominant peak at *m*/*z* = 4444.69 corresponding to [Ag_40_(C_2_B_10_H_10_S_2_)_12_(PPh_3_)_8_]^2^^−^ The peaks at *m*/*z* = 4313.65 and *m*/*z* = 4182.15 could be assigned to the [Ag_40_(C_2_B_10_H_10_S_2_)_12_(PPh_3_)_7_]^2^^−^ and [Ag_40_(C_2_B_10_H_10_S_2_)_12_(PPh_3_)_6_]^2^^−^ species, respectively, which stems from the dissociation of the PPh_3_ ligands in the ligand shell (**Figure** **4**a). It is worth noting that the dissociation of weakly coordinated PPh_3_ from metal NC during ionization was commonly observed.^[^ ^51^, ^52^ ^]^ The Ag_12_ NC in **Ag_40_@Ag_12_** was not detected in the MS spectrum as in **Ag_12_**, probably attributed to its charge neutrality. UV–vis absorption spectroscopy was also employed to investigate the constituents of **Ag_40_@Ag_12_** (Figure 4b). The normalized UV–vis absorption spectra of **Ag_12_**, **Ag_40_**, and **Ag_40_@Ag_12_** in solution demonstrate that the Ag_12_ NCs only presented a peak at ≈267 nm, which could be ascribed to the π–π* transitions of PPh_3_ and *m*‐carboranedithiols ligands, while **Ag_40_@Ag_12_** showed extra characteristic absorption at 356, 425, and 618 nm, which were as alike as **Ag_40_**, confirming the co‐existence of the Ag_12_ and Ag_40_ NCs. **Ag_40_@Ag_12_** showed similar XPS results as **Ag_40_**, indicating the existence of Ag(I) and Ag(0) (Figure S10, Supporting Information).

To figure out the existence form of the Ag_40_ NCs in **Ag_40_@Ag_12_**, UV–vis diffuse reflectance spectra were recorded (Figure 4c). The crystalline **Ag_12_** displayed a major absorption band before 500 nm with a distinct peak at ≈305 nm, which undergoes a significant redshift in comparison with the absorption band in solution. The **Ag_40_** crystals presented a broad successive absorption from 250 to 1300 nm instead of the molecular absorption spectra in the solution. The redshift and broadening of the reflectance spectra could be ascribed to the ordered packing and electronic coupling of the metal NCs. In comparison, the crystalline **Ag_40_@Ag_12_** showed two distinct absorption peaks at 305 and 430 nm but with an absorption edge extending over that of **Ag_40_**, which could be ascribed to charge‐transfer transitions between the Ag_40_ and Ag_12_ NCs.^[^ ^53^ ^]^ The absorption bands at 305 and 430 nm could be assigned to the characteristic absorption of Ag_12_ and Ag_40_, respectively. Notably, the absorption band at 430 nm shows a combined feature of the Ag_40_ NCs in crystalline and solution phases. The conservative redshift and broadening of the characteristic absorption bands corresponding to the Ag_40_ NCs in crystalline **Ag_40_@Ag_12_** suggested that the interactions between the transition dipole moment of the individual absorbing Ag_40_ NCs and the induced dipole moments in the adjacent Ag_40_ NCs are weak.^[^ ^54^ ^]^ In other words, the electronic coupling between the Ag_40_ NCs in crystalline **Ag_40_@Ag_12_** is less than that in **Ag_40_**. In addition, PXRD analysis did not detect the presence of crystalline Ag_40_ (Figure 4d). Combining the XRD and solid‐state UV absorption results, we proposed that the Ag_40_ NCs confined inside the **Ag_40_@Ag_12_** crystals were in an amorphous state. The lattice mismatch of the **Ag_12_** and **Ag_40_** also suggested that the Ag_40_ could only be amorphous within the **Ag_40_@Ag_12_**.

**Figure 5:**
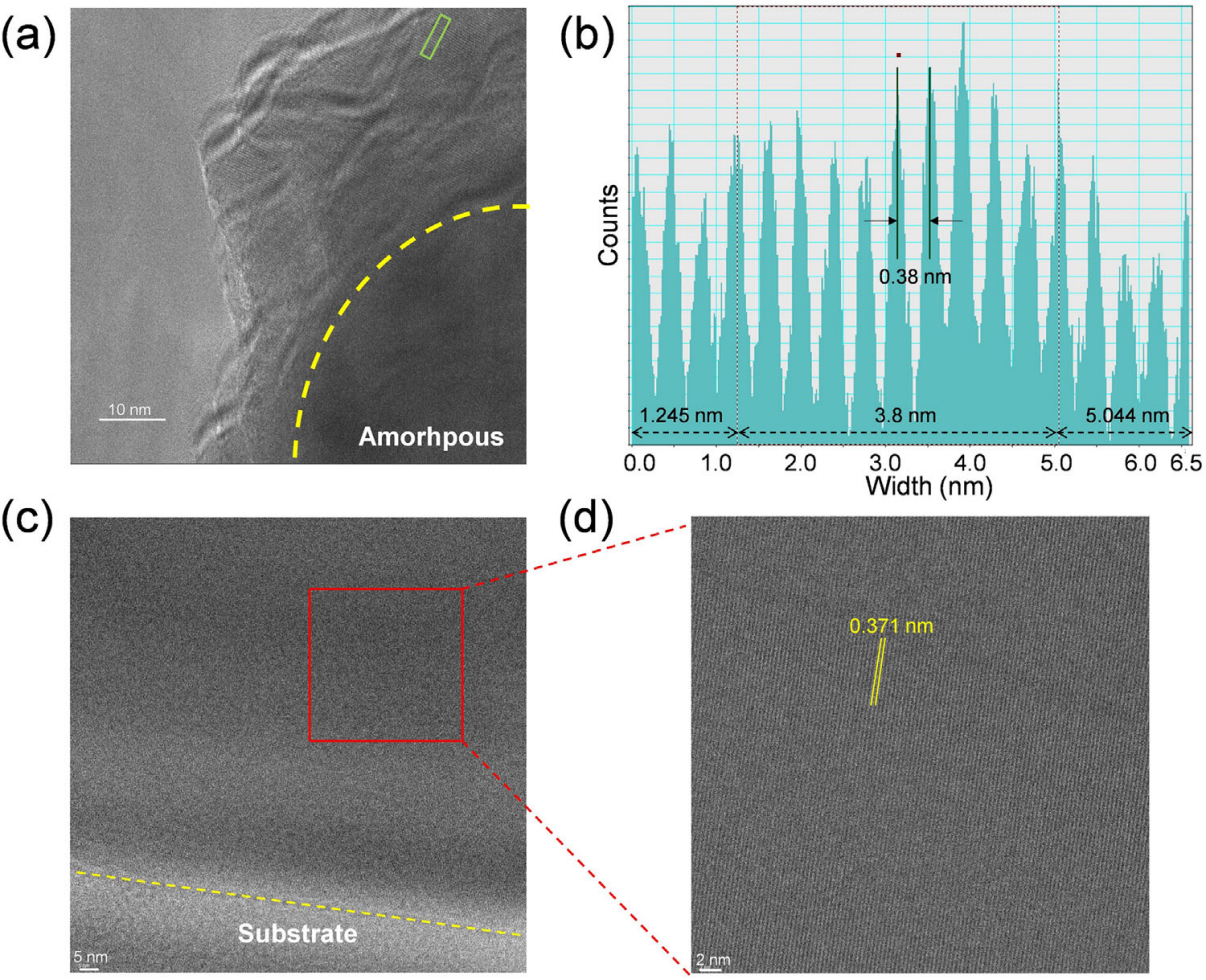
a) Cryo‐TEM image of the **Ag_40_@Ag_12_** nanosheet, the interfaces between crystalline Ag_12_ and amorphous Ag_40_ are marked with yellow dashed lines. b) Profiles inside the rectangular area of the cryo‐TEM image. c) Cryo‐TEM image of **Ag_12_** nanosheet. d) Cryo‐TEM image of **Ag_12_** nanosheet on a large scale.

To further confirm the crystalline‐amorphous heterostructure of Ag_40_@Ag_12_, cryo‐transmission electron microscope (cryo‐TEM) was conducted. First, the **Ag_40_@Ag_12_** crystals were sectioned with an ultramicrotome to afford nanosheets with a thickness of 50–70 nm. Then, the nanosheets with exposed Ag_40_ NCs were transferred for cryo‐TEM measurement. The cryo‐TEM image shows that the nanosheets have well‐defined interfaces between crystalline and amorphous regions (**Figure** **5**a). The crystalline region shows distinct lattice fringes, and the interplanar spacing is determined to be 3.80 Å, which is similar to that of pure **Ag_12_** crystals‐based nanosheets (Figure 5b–d). These results collectively demonstrated that the Ag_40_ NCs lost the long‐range ordered arrangement and were confined into the **Ag_12_** crystals.

### 2.3 Deduction on the Formation Mechanism of the Ag_40_@Ag_12_

**Figure 6:**
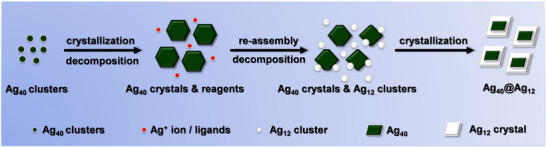
Speculation on the formation mechanism of Ag_40_@Ag_12_.

Considering the time‐dependent UV–vis absorption along with the crystallization progress of the NCs, we speculate that the formation mechanism of the heterostructure **Ag_40_@Ag_12_** with special morphology involves three stages (**Figure** **6**). First, the Ag_40_ NCs crystallized in microcrystals as nuclei. In this process, the Ag_40_ NCs in DMF would also decompose gradually, as verified by the time‐dependent UV–vis absorption (Figure S13, Supporting Information), and the dissociation could be inhibited by adding additional PPh_3_ (Figure S21, Supporting Information). In the second stage, with the starting materials provided from the dissociation of the Ag_40_ NCs, the Ag_12_ NCs were assembled. The concentration of the Ag_12_ NCs in the solution would increase gradually as the Ag_40_ crystals decompose simultaneously. In the third stage, the Ag_12_ NCs undergo epitaxially growth to encapsulate the Ag_40_ seeds, resulting in the **Ag_40_@Ag_12_** heterostructure. The crystals of **Ag_40_** lose their original shape and become an amorphous state upon etching by the peripheral Ag_12_ NCs. Another critical issue is the reducing agent. The addition of a larger amount of NaBH_4_ (8 mg) could inhibit the direct assembly of **Ag_12_**, and the high concentration of Ag_40_ NCs led to the crystallization of **Ag_40_** as the seed crystals. By contrast, the yield of the Ag_12_ NCs would be comparable to the Ag_40_ NCs with a smaller amount of NaBH_4_ (4 mg), therefore, resulting in the synchronous crystallization of **Ag_12_** and **Ag_40_**.

### 2.4 Photothermal Properties

The core–shell structures potentially have better photothermal properties.^[^ ^55^, ^56^ ^]^ Thus, we explore the potential photothermal conversion of **Ag_12_**, **Ag_40_**, and **Ag_40_@Ag_12_**. Under the 808 nm laser irradiation at the power density of 200 mW cm^−2^, the temperature of these samples increased rapidly and reached 28.6, 118.1, and 123.6 °C, respectively, after 2 min of irradiation, indicating the superior photothermal effect of **Ag_40_@Ag_12_** is comparison to **Ag_12_** and **Ag_40_** (Figure S22a,b, Supporting Information). The **Ag_12_** shell in **Ag_40_@Ag_12_** acts as a protective layer to reduce the heat loss,^[^ ^57^, ^58^ ^]^ meanwhile, potentially promoting light scattering and internal reflections,^[^ ^59^ ^]^ which in turn boosts the photothermal conversion efficiency of the inner Ag_40_ nanoclusters. Additionally, **Ag@Ag_12_** shows good photothermal stability, maintaining intact after five cycles of laser on/off. As shown in Figure S22c,d (Supporting Information), the temperature of **Ag_40_@Ag_12_** is positively related to the power density.

## 3 Conclusion

In summary, an amorphous‐crystalline heterostructured **Ag_40_@Ag_12_** containing two novel carboranylthiolate‐protected silver clusters Ag^I^_12_ and Ag_40_ was constructed. The Ag_12_ NC features a 3D metallosupramolecular cage based on cluster nodes, and the Ag_40_ NC is superatomic with a three‐shell Platonic metal framework. Further investigation of **Ag_40_@Ag_12_** suggests that the amorphous Ag_40_ NCs are encapsulated by crystalline **Ag_12_**, and the formation of **Ag_40_@Ag_12_** involves processes including the crystallization of **Ag_40_** →partial decomposition of the Ag_40_ NCs and formation of the Ag_12_ NCs→recrystallization of **Ag_12_**. Heterostructural materials integrate the properties of multiple components, and the synergistic effect derived from the components can expand their potential applications, including in energy storage, sensing, and catalysis. In the case of Ag_40_@Ag_12_ reported here, Ag_40_ is metastable (sensitive to O_2_ and H_2_O) while Ag_12_ is ultra‐stable. Thus, Ag_12_ as a shield could prevent Ag_40_ from direct exposure to O_2_ and H_2_O, providing a new pathway for stabilizing and storing metastable metal nanoclusters. The present study introduces a novel class of heterostructured materials and provides implications for extending the macroscopic co‐assembly of distinctive silver clusters.

[CCDC 2322648, 2322649, and 2322650 contain the supplementary crystallographic data for this paper. These data can be obtained free of charge from The Cambridge Crystallographic Data Centre via www.ccdc.cam.ac.uk/data_request/cif.]

## Conflict of Interest

The authors declare no conflict of interest.

## Supporting information



Supporting Information

## Data Availability

The data that support the findings of this study are available from the corresponding author upon reasonable request.
