# Supplementary material for: Crystalline‐Amorphous Heterostructure: A Novel Configuration for Silver Nanoclusters
Source: Adv Sci (Weinh). 2025 Jun 29;12(33):e01186. doi: 10.1002/advs.202501186 (PMC12412518; doi:10.1002/advs.202501186)
Supplement: Supplementary file 1 — Supporting Information [file ADVS-12-e01186-s001.docx]

Supporting Information

Crystalline-Amorphous Heterostructure: A Novel Configuration for Silver Nanoclusters

Jia-Hong Huang,^1^ Yao Cui,^1^ Peng Luo,^2^ Zhao-Yang Wang,^*1^ and Shuang-Quan Zang^*1^

1 College of Chemistry, Zhengzhou University, Zhengzhou 450001, China.

2 College of Chemistry and Chemical Engineering, Henan Polytechnic University, Jiaozuo 454000, China.

*E-mail: [wangzy@zzu.edu.cn](mailto:wangzy@zzu.edu.cn)

[zangsqzg@zzu.edu.cn](mailto:zangsqzg@zzu.edu.cn)

Section S1: Materials and Methods

**Materials**

AgNO_3_, triphenylphosphine (PPh_3_), sodium borohydride (NaBH_4_, 98.0%) were purchased from Aladdin. All reagents and solvents were used directly as received without further purification. 1,7-Bis(mercapto)-*m*-carborane was prepared according to the literature method.^[1](#_ENREF_1)^

**Instrumentation.**

^1^H NMR and ^13^C NMR spectra were recorded on a Bruker DRX spectrometer operating at 400 MHz. X-ray photoelectron spectroscopy (XPS) was conducted using a Thermo ESCALAB 250XI with Al Kα radiation as the excitation source. Binding energies were calibrated by C 1s at 284.8 eV. Electrospray ionization mass spectrometry (ESI-MS) was performed on a X500R QTOF spectrometer. UV/Vis absorption spectra were recorded in the range of 200-800 nm using an U-2000 UV-visible spectrophotometer. Solid UV-Vis diffuse reflectance spectra were recorded with Hitachi UH4150 spectrometer. Powder X-ray diffraction (PXRD) were collected using a Rigaku MiniFlex600 diffractometer (Cu Kα, *λ* = 1.54178 Å). Fourier transform infrared (FT-IR) spectra were recorded on a Bruker ALPHA II spectrometer. The Ag_40_@Ag_12_ sections were prepared by Leica EM UC7 Ultramicrotome. Cyro-TEM was performed using Thermo Scientific Glacios 2 Cryo-Transmission Electron Microscope (cryo-TEM) operated at 200 kV, which is equipped with automatic injection system of frozen sample.

**Single-Crystal X-ray Diffraction Analysis**.

The diffraction data were collected on a Rigaku XtaLAB Pro diffractometer with Cu-Kα radiation (*λ* = 1.54184 Å). Data collection and reduction were performed using the program CrysAlisPro.^2^ The structure was solved with direct methods (*SHELXS-2015*)^3^ and refined using full-matrix least-squares based on *F*2 with the programs SHELXS-97 and SHELXL-97 within *OLEX2*.^4^ There was a large solvent-accessible void volume in the crystals of Ag_40_, which were occupied by highly disordered solvent molecules. All nonhydrogen atoms were refined anisotropically, and the hydrogen atoms were included at idealized positions. The SQUEEZE option of PLATON was used at the final refinement to account for the contribution of disordered solvent molecules to the calculated structure factors.^5^ The crystal structures are visualized by DIAMOND 3.2.^6^ Detailed information on the crystal data, data collection, and refinement data for Ag_12_, Ag_40_, and Ag_40_@Ag_12_ are provided in Table S1.

Section S2: Synthesis

**Synthesis of** **Ag_12_(C_2_B_10_H_10_S_2_)_6_(PPh_3_)_4_.**

First, 1,7-bis(mercapto)-*m*-carborane (4.2 mg, 0.02 mmol) and AgNO_3_ (6.8 mg, 0.04 mmol) were dissolved in 4.5 mL mixed solvent of MeOH-CH_2_Cl_2_ (2:1 v/v). After PPh_3_ (6mg, 0.023 mmol) was added, the turbid solution turned clear within several minutes. The colorless cubic crystals were formed within 1 day of evaporation. Collective yield: *approx*. 80.0% (based on Ag).

**Synthesis of Ag_40_(C_2_B_10_H_10_S_2_)_12_(PPh_3_)_8_.**

First, 1,7-bis(mercapto)-*m*-carborane (4.2 mg, 0.02 mmol) and AgNO_3_ (6.8 mg, 0.04 mmol) were dissolved in 4.5 mL mixed solvent of MeOH-CH_2_Cl_2_ (2:1 v/v). After PPh_3_ (6mg, 0.023 mmol) was added, the turbid solution turned into clear within several minutes. To this solution, 4.0 mg (0.1 mmol) of aqueous NaBH_4_ (500 μL) was added dropwise under vigorous stirring. The resulting solution color changed from light yellow to transparent dark red. The reaction was allowed to stand for 4 h in the absence of light. The resultant dark precipitate was washed with methanol three times and dissolved in DMF solution. The black hexagonal crystals of Ag_40_ and colorless cubic crystals of Ag_12_ were formed within two weeks of evaporation. The pure phase Ag_40_ crystals could be obtained by diffusing diethyl ether into the DMF solution because this method isolates oxygen, preventing the degradation of Ag_40_. Collective yield: *approx*. 30.0% (based on Ag).

**Synthesis of Ag_40_@Ag_12_.**

The following procedures were kept unchanged according to the synthesis of Ag_40_(C_2_B_10_H_10_S_2_)_12_(PPh_3_)_8_, but the addition of NaBH_4_ was increased to 8 mg.

**Explanation of the different NaBH_4_ amount results in Ag_40_ and Ag_40_@Ag_12_**

**Ag_40_** is a Ag(0)-containing superatomic nanocluster. NaBH_4_ as a reducing agent is indispensable for constructing those nanoclusters containing free electrons. By comparison, **Ag_12_** is a Ag(I) nanocluster that could be synthesized in the absence of the reducing agent. **Ag_12_**, **Ag_40_**, and **Ag_40_@Ag_12_** were synthesized under the same reaction conditions (ligand, metal ion, and solution) except for the amount of NaBH_4_. If the NaBH_4_ is less than 4 mg, only partial Ag(I) could be reduced to Ag(0). In addition, the decomposition of **Ag_40_** also releases the Ag(I). The high Ag(I) concentration facilitates the **Ag_12_** assembly, resulting in the synchronous crystallization of **Ag_12_** and **Ag_40_**. With the amount of NaBH_4_ increased to 8 mg, massive Ag(I) was reduced to Ag(0), facilitating the **Ag_40_** assembly. Thus, the **Ag_40_** crystallized in microcrystals first. After the partial **Ag_40_** nanocluster was oxidized and decomposed, the released Ag(I) facilitated the **Ag_12_** assembly and underwent epitaxial growth on the **Ag_40_** seeds.

Section S4 Theoretical calculations

**Calculations of the theoretical structure of Ag_40_**

The calculations were performed using the semiempirical quantum mechanical methods GFN1-xTB packages^7-9^. The single crystal structure was chosen as the initial guess for ground state optimization at tight level. The optimized structure of the clusters preserved the basic characteristics of the input structure, only with slight changes in the bond length, bond angles, and dihedral angles, which confirming the feasibility of GFN1-xTB methods for cluster calculations. The UV-Vis spectra were calculated by sTDA^10-11^.

Section S3 Characterizations


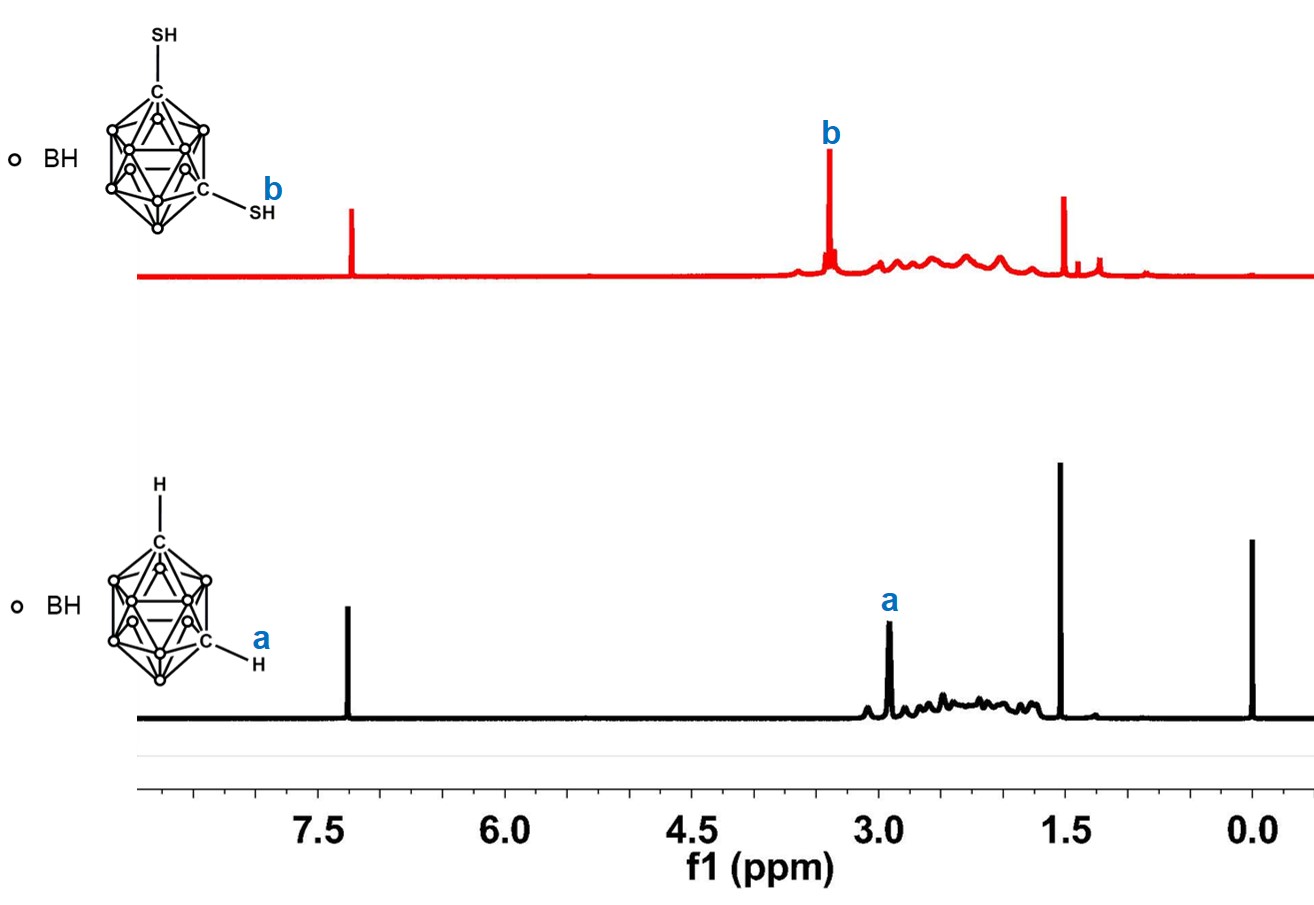


**Figure S1.** ^1^H-NMR (CDCl_3_) spectra of 1,7-bis(mercapto)-*m*-carborane (blue line) and 1, 7-*m*-carborane (red line)


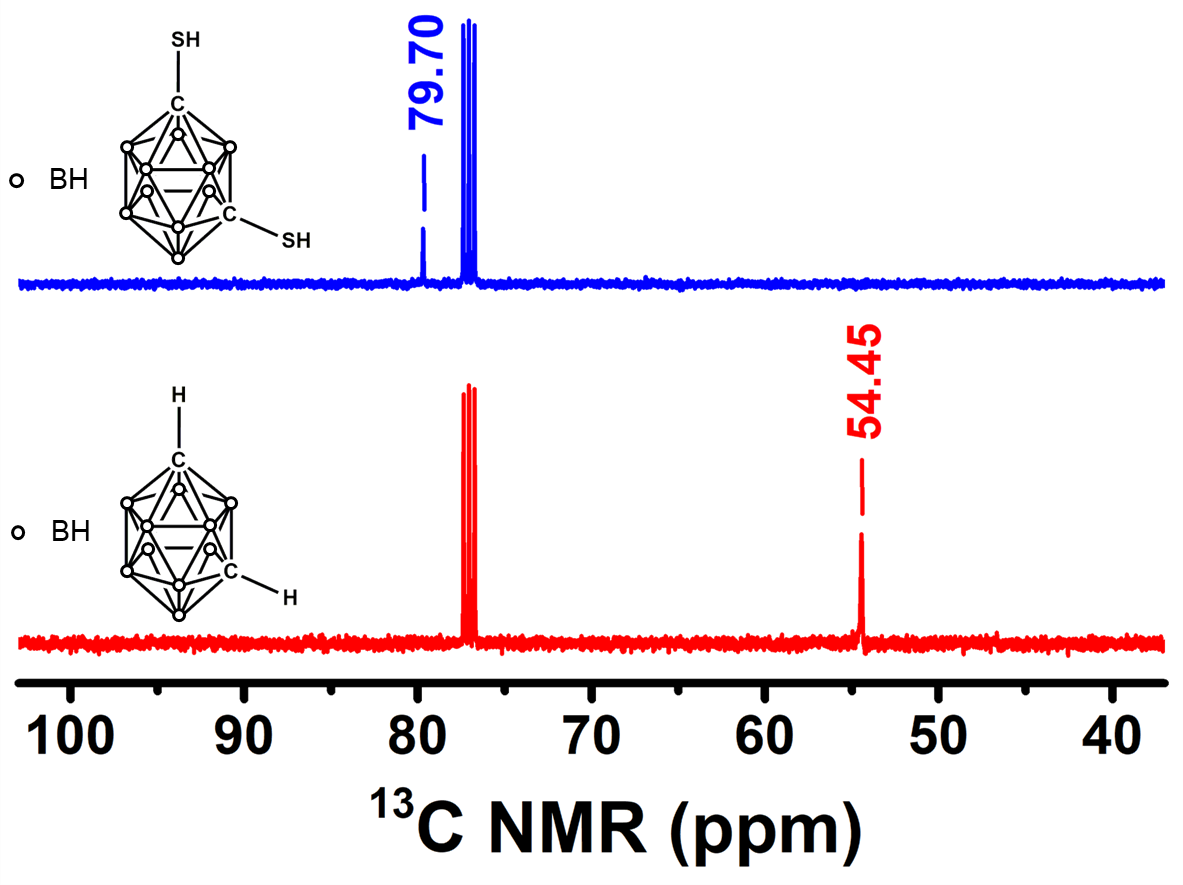


**Figure S2.** ^13^C-NMR (CDCl_3_) spectra of 1,7-bis(mercapto)-*m*-carborane (blue line) and 1, 7-*m*-carborane (red line)


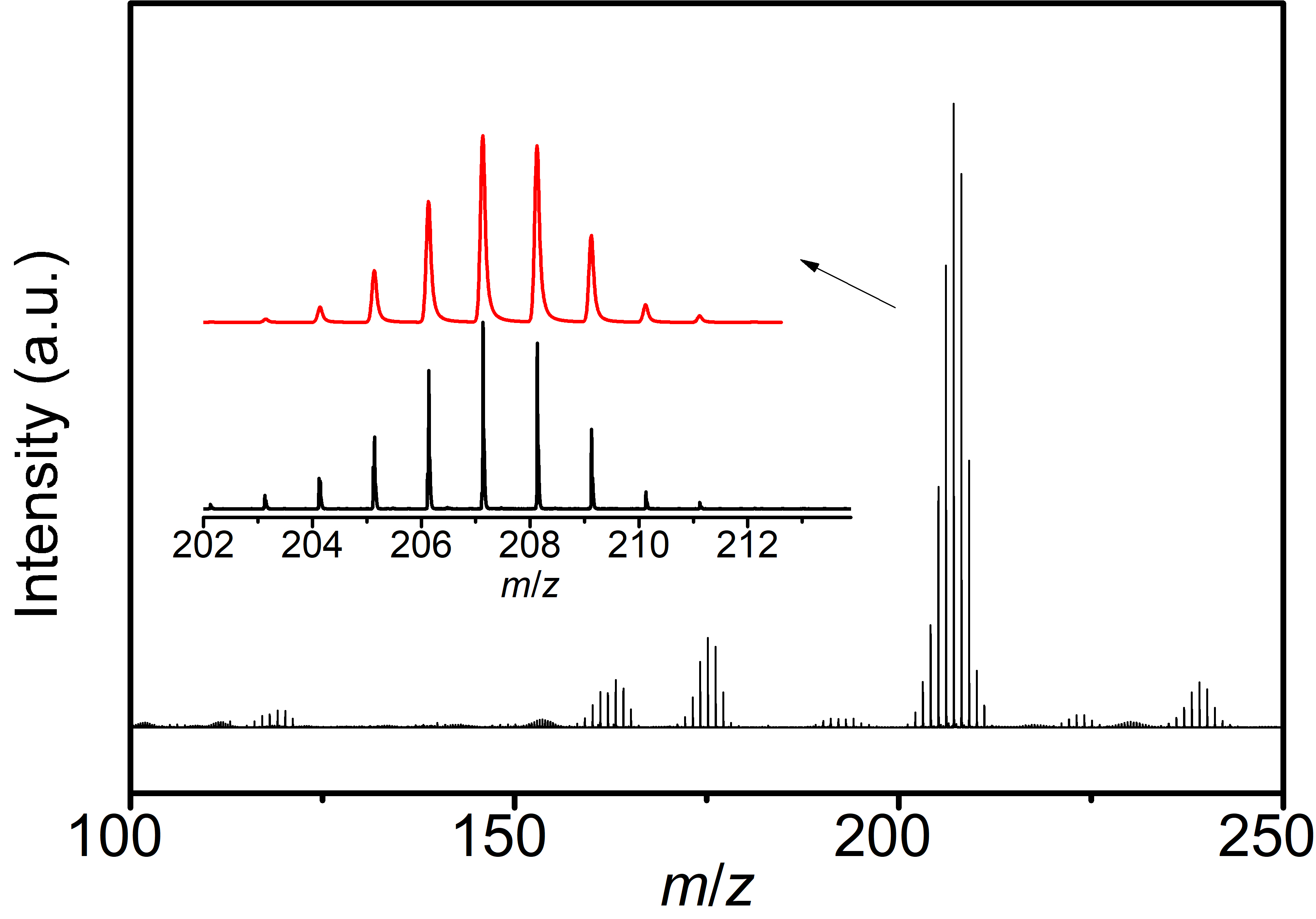


**Figure S3.** Negative-mode ESI-MS spectrum of 1,7-bis(mercapto)-*m*-carborane.


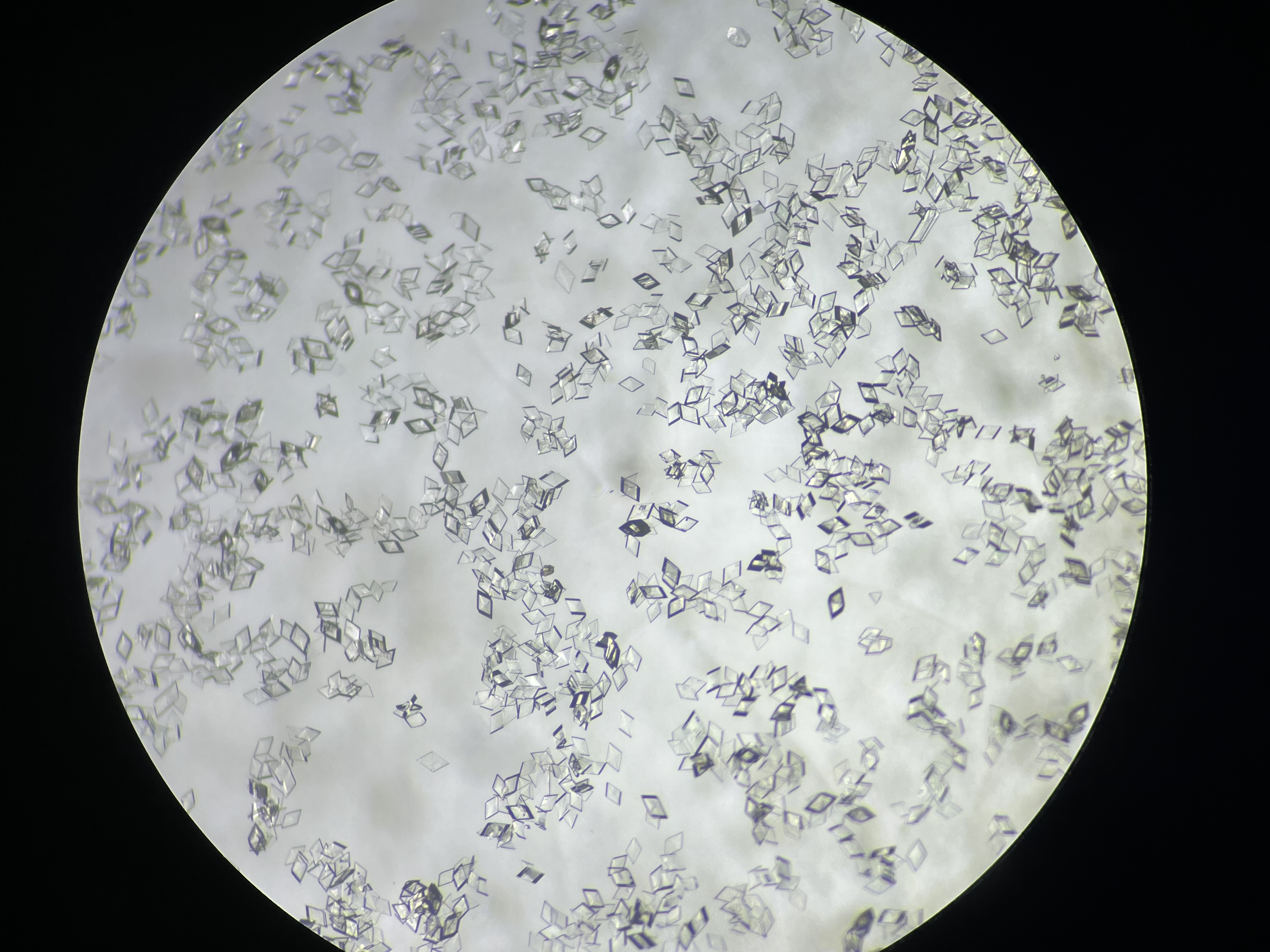


**Figure S4.** Images of **Ag_12_** crystals.


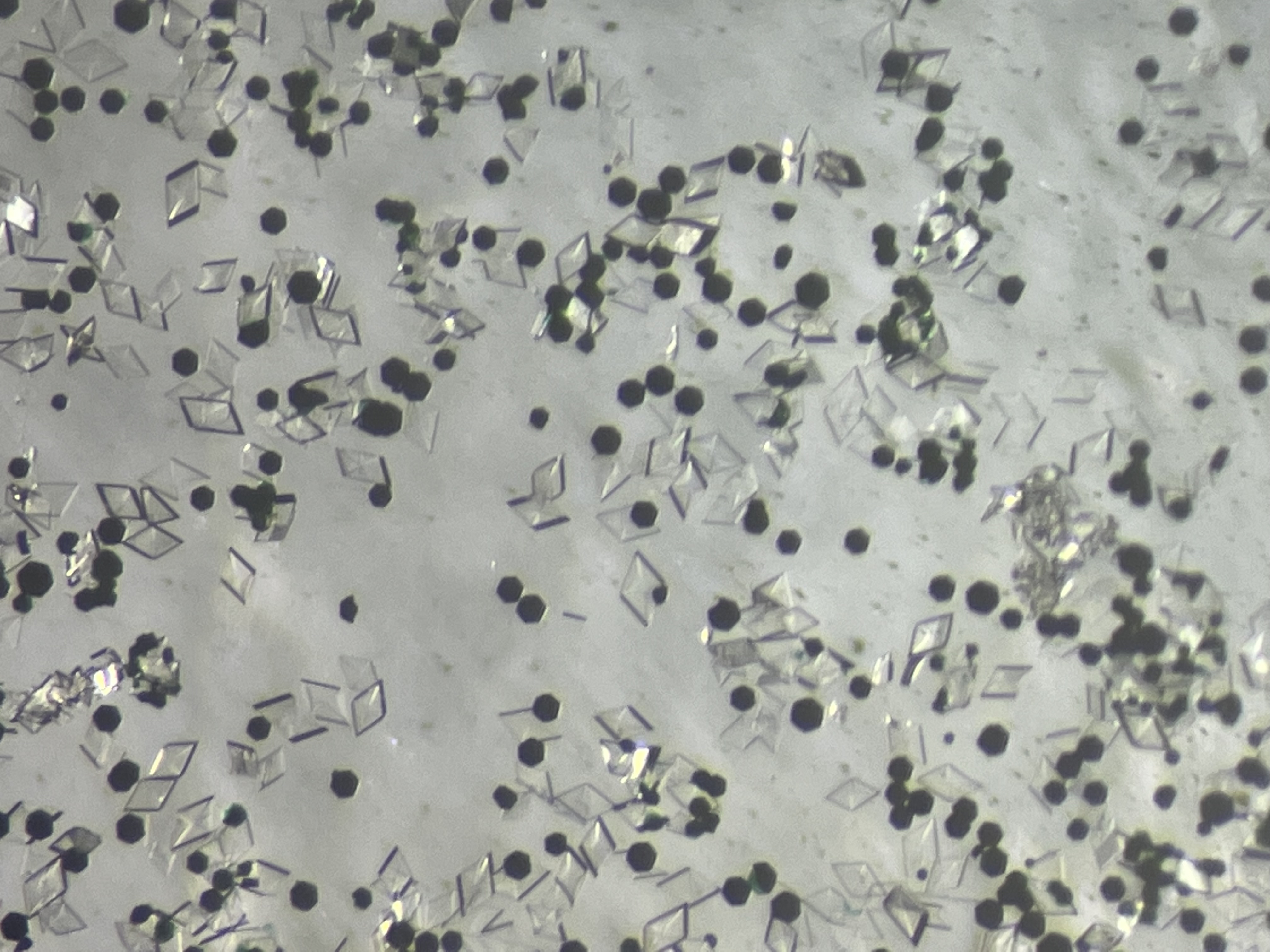


**Figure S5.** Images of the mixture of Ag_12_ (rhombic) and Ag_40_ (hexagon) crystals.


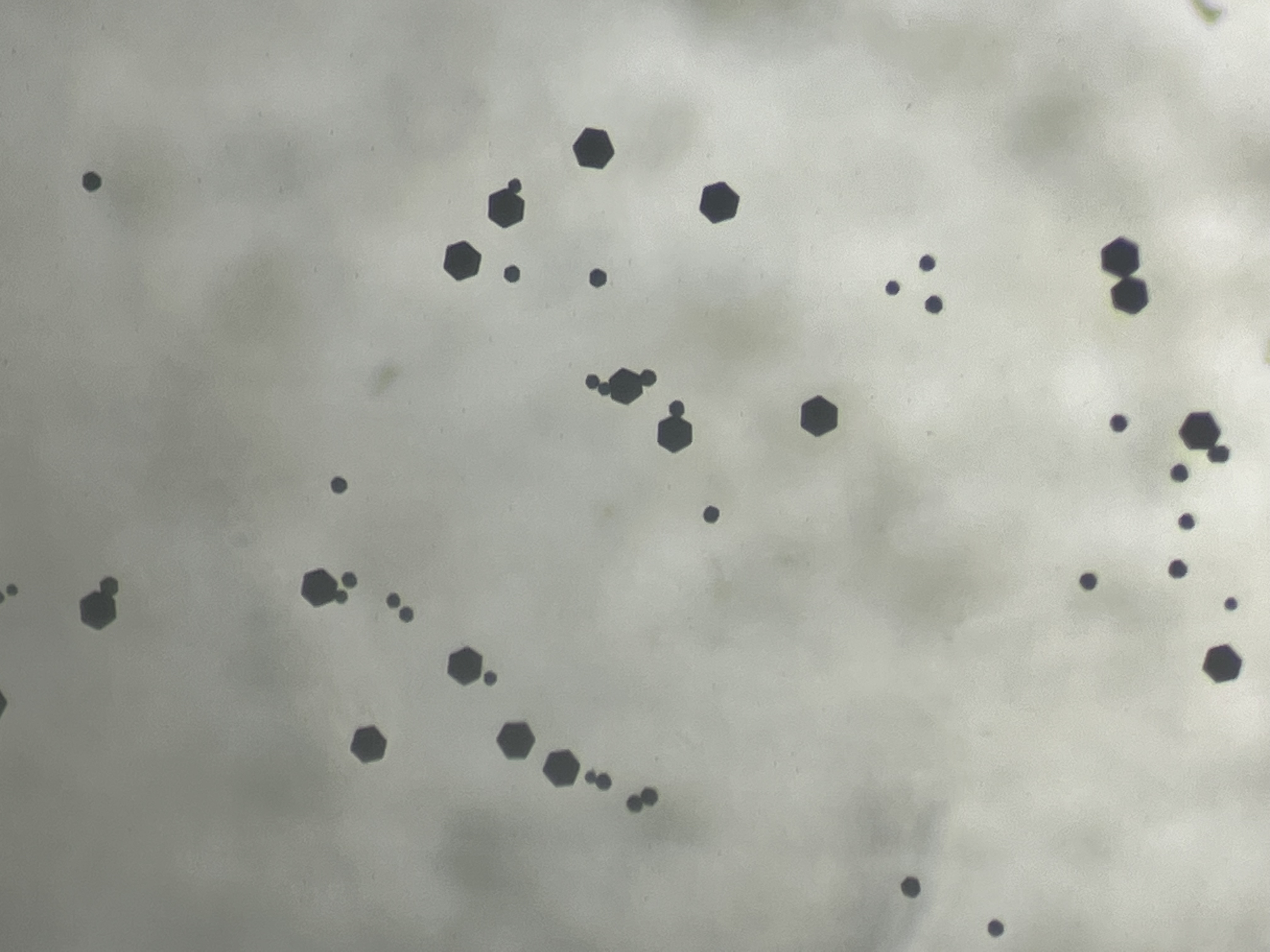


**Figure S6.** Images of Ag_40_ crystals.


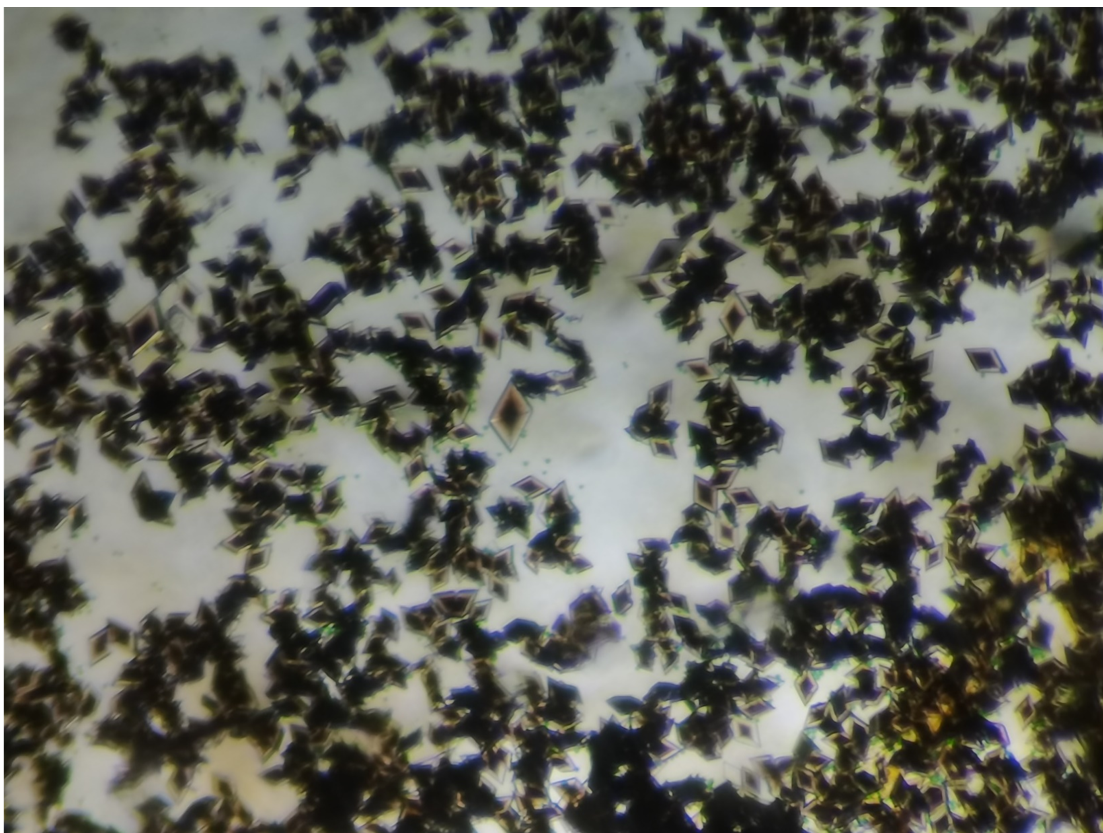


**Figure S7.** Images of Ag_40_@Ag_12_ crystals.


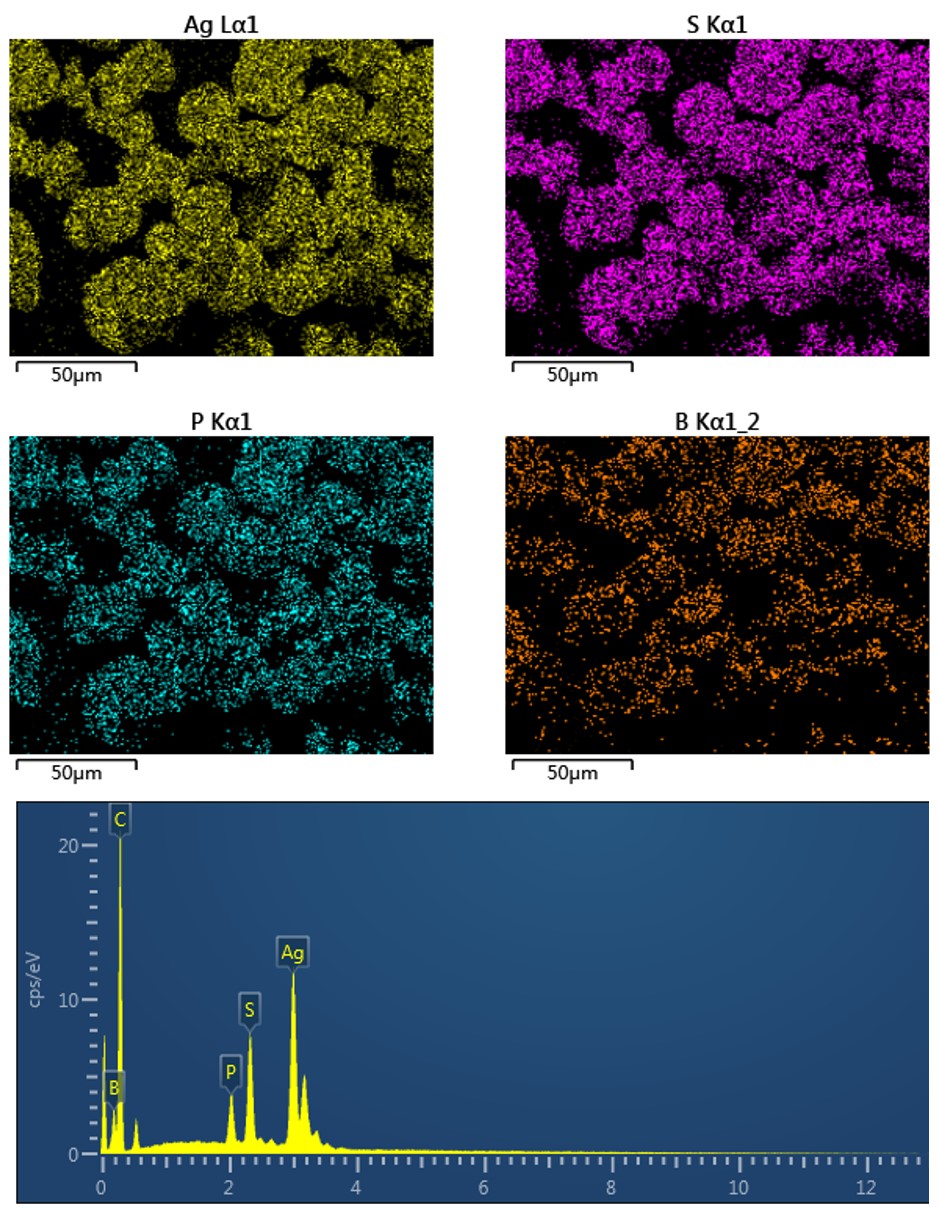


**Figure S8.** Energy dispersive spectroscopy (EDS) mapping results of the Ag_40_.


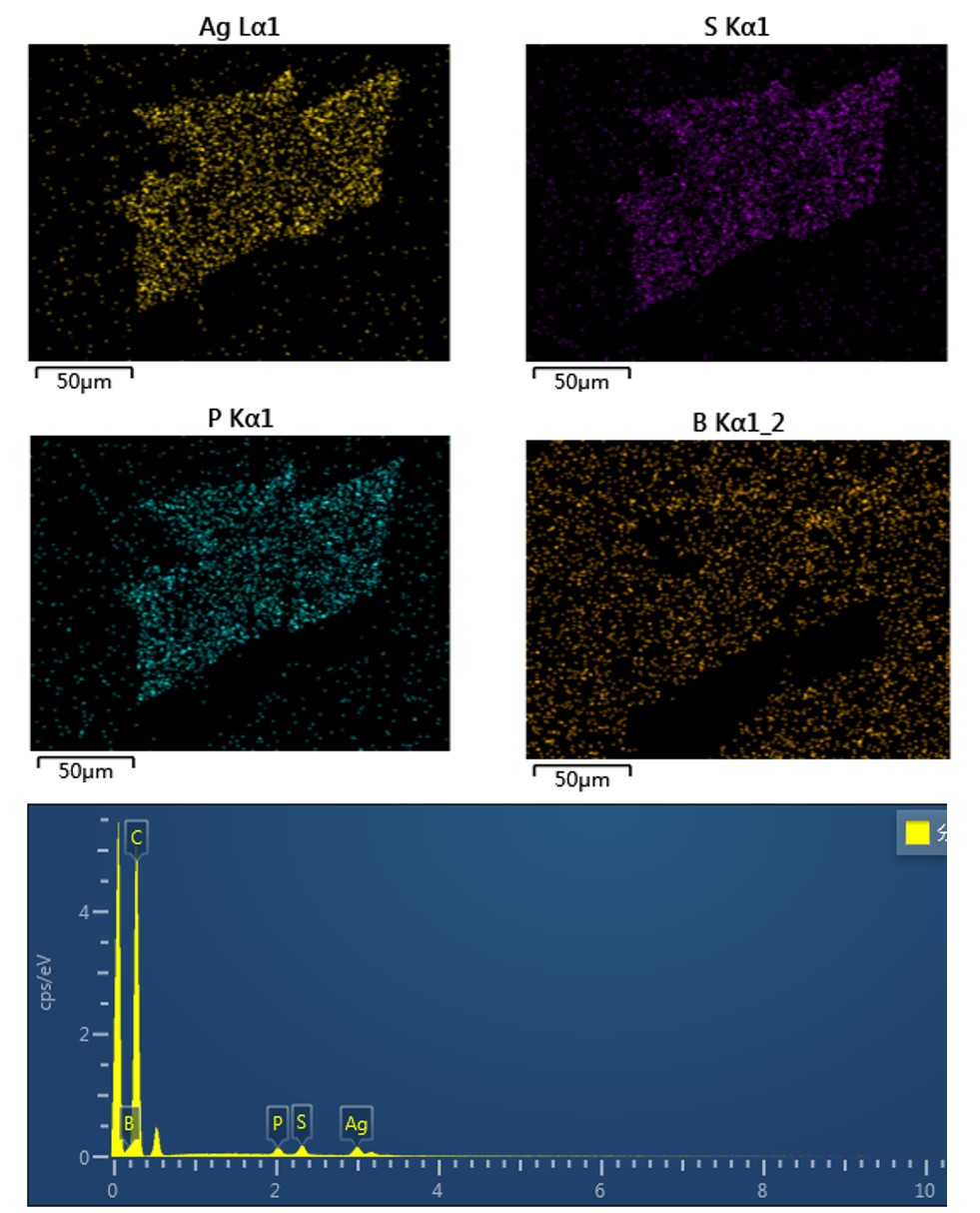


**Figure S9.** Energy dispersive spectroscopy (EDS) mapping results of the Ag_40_@Ag_12_.


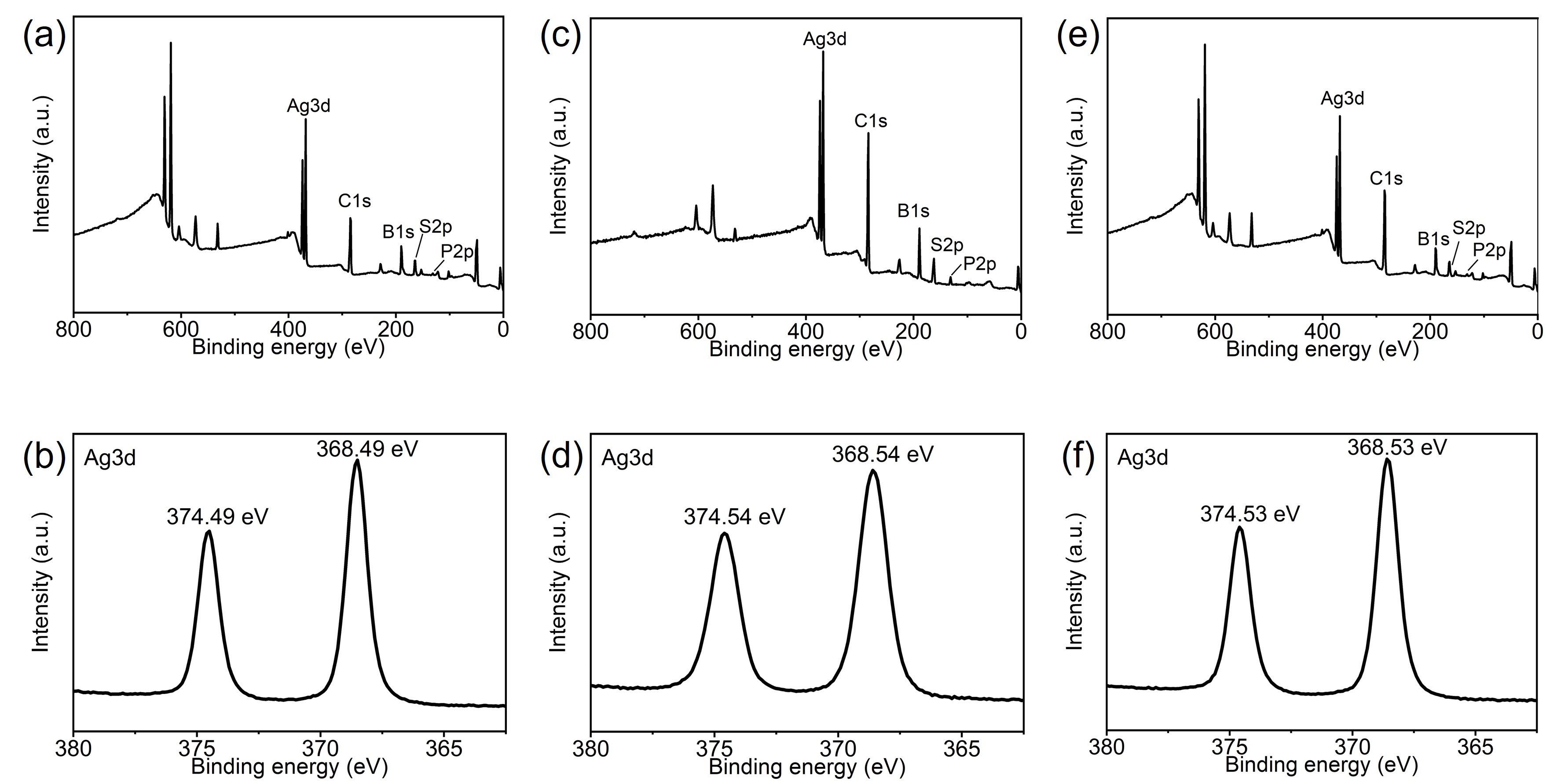


**Figure S10.** (a) XPS survey scan of Ag_12_. (b) Ag 3d XPS spectra of Ag_12_. (c) XPS survey scan of Ag_40_. (d) Ag 3d XPS spectra of Ag_40_. (e) Ag 3d XPS spectra of Ag_40_@Ag_12_. (f) Ag 3d XPS spectra of Ag_40_@Ag_12_.


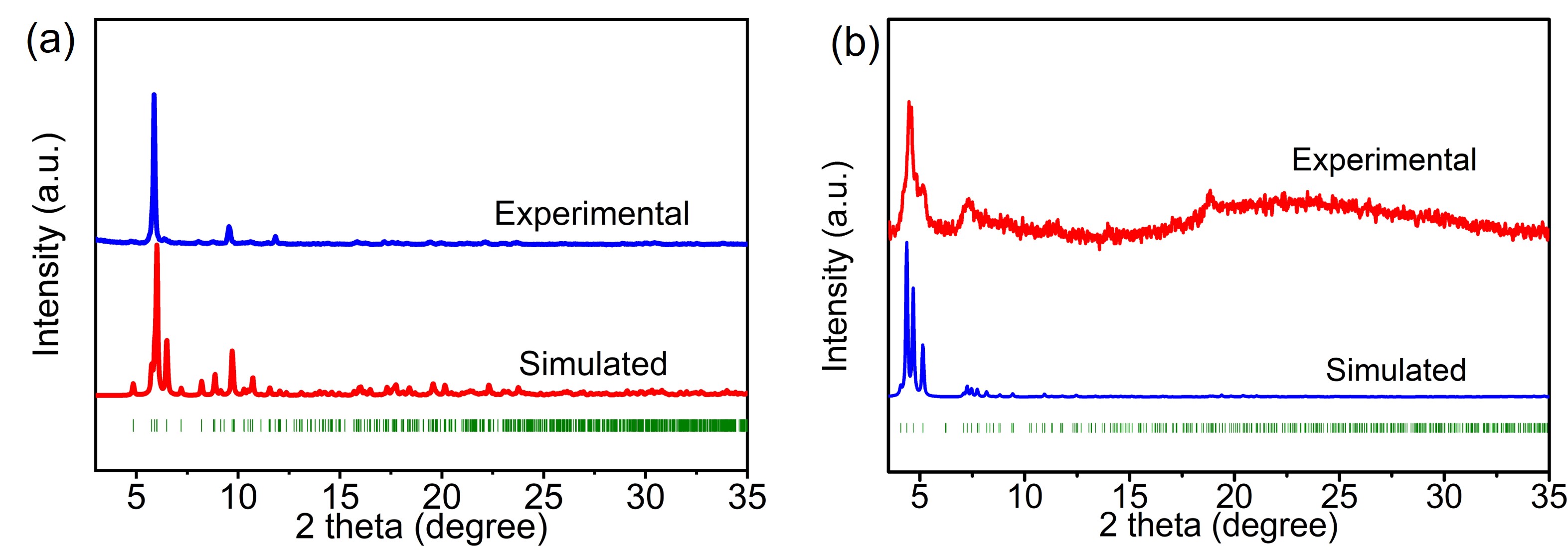


**Figure S11.** PXRD patterns of **Ag_12_** (a) and **Ag_40_** (b) crystals.


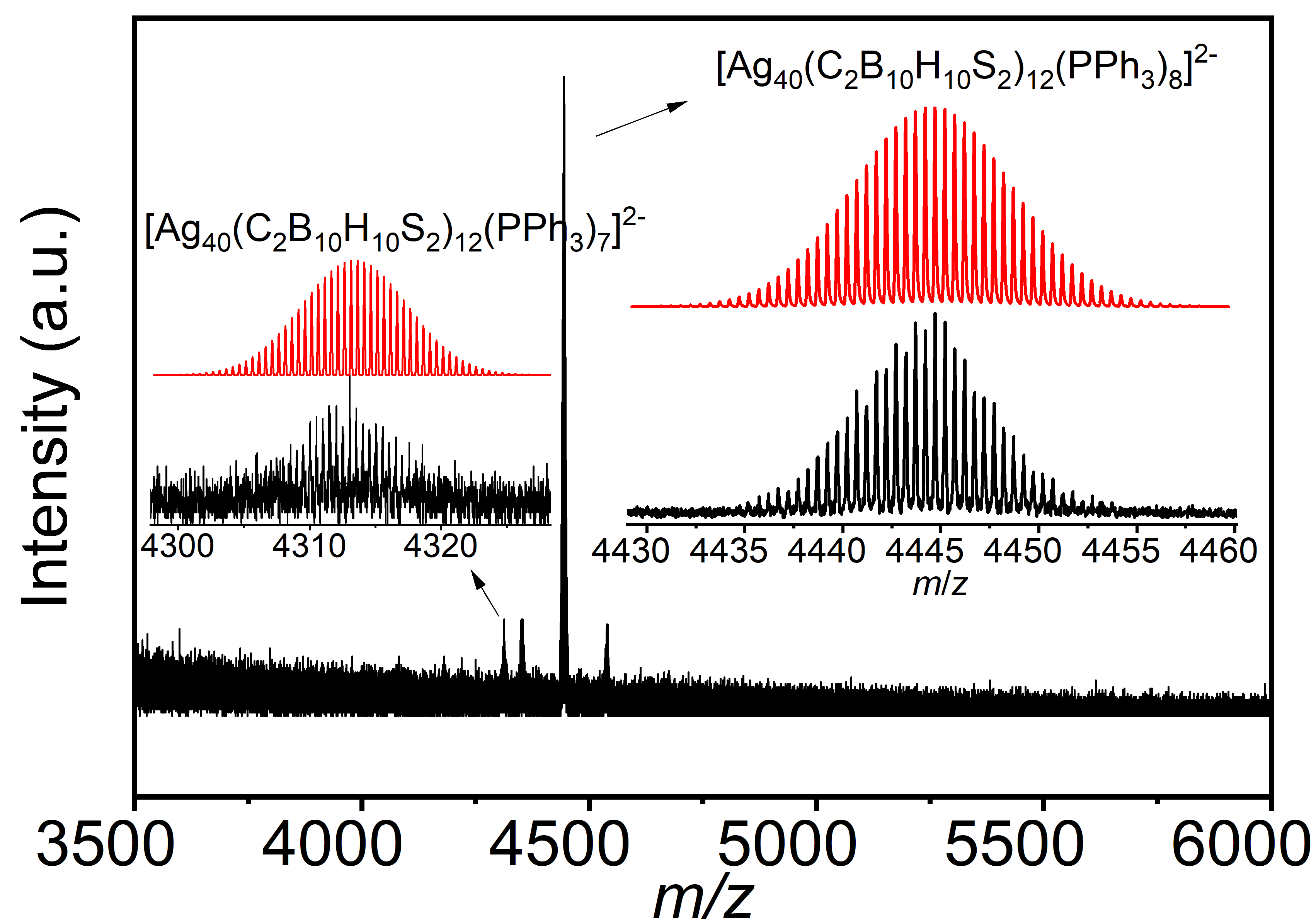


**Figure S12.** Negative-mode ESI-MS spectrum of **Ag_40_**. Inset: The enlarged portion of the spectrum showed the measured (black) and simulated (red) isotopic distribution patterns.


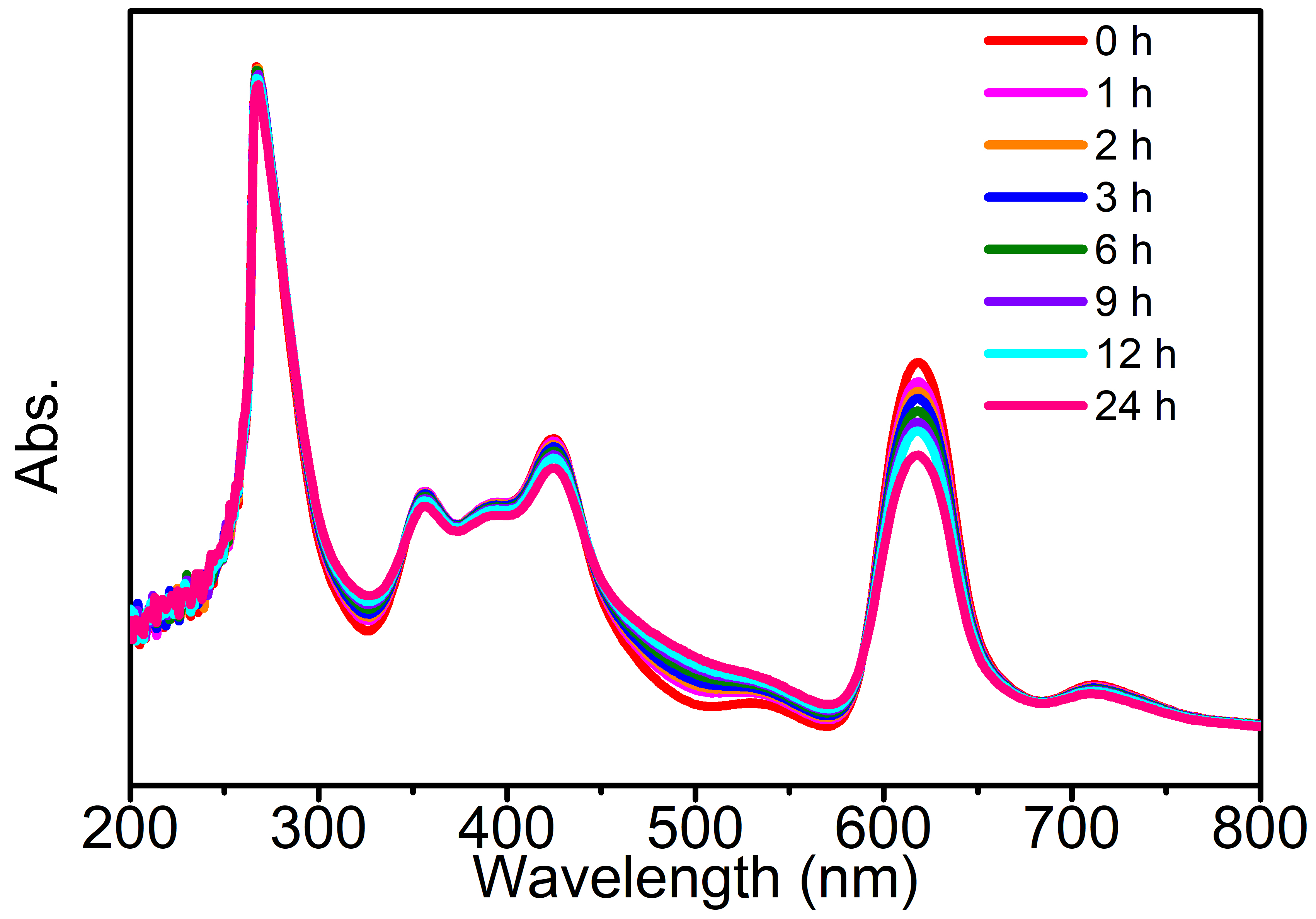


**Figure S13.** Time-dependent UV-vis absorption spectra of **Ag_40_**.


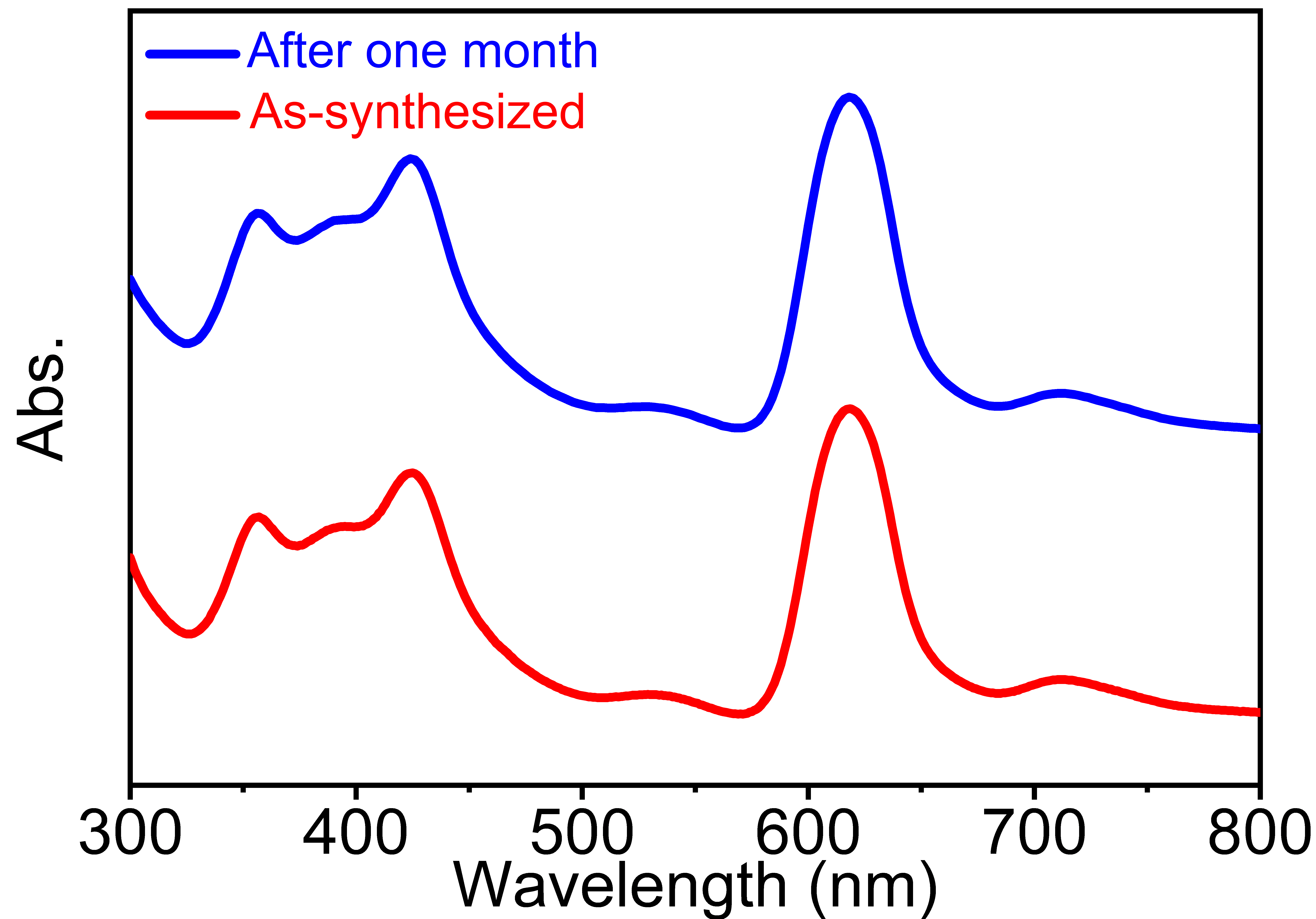


**Figure S14.** UV–vis absorption spectra of as-synthesized Ag_40_ nanocrystals and after one month of storage.


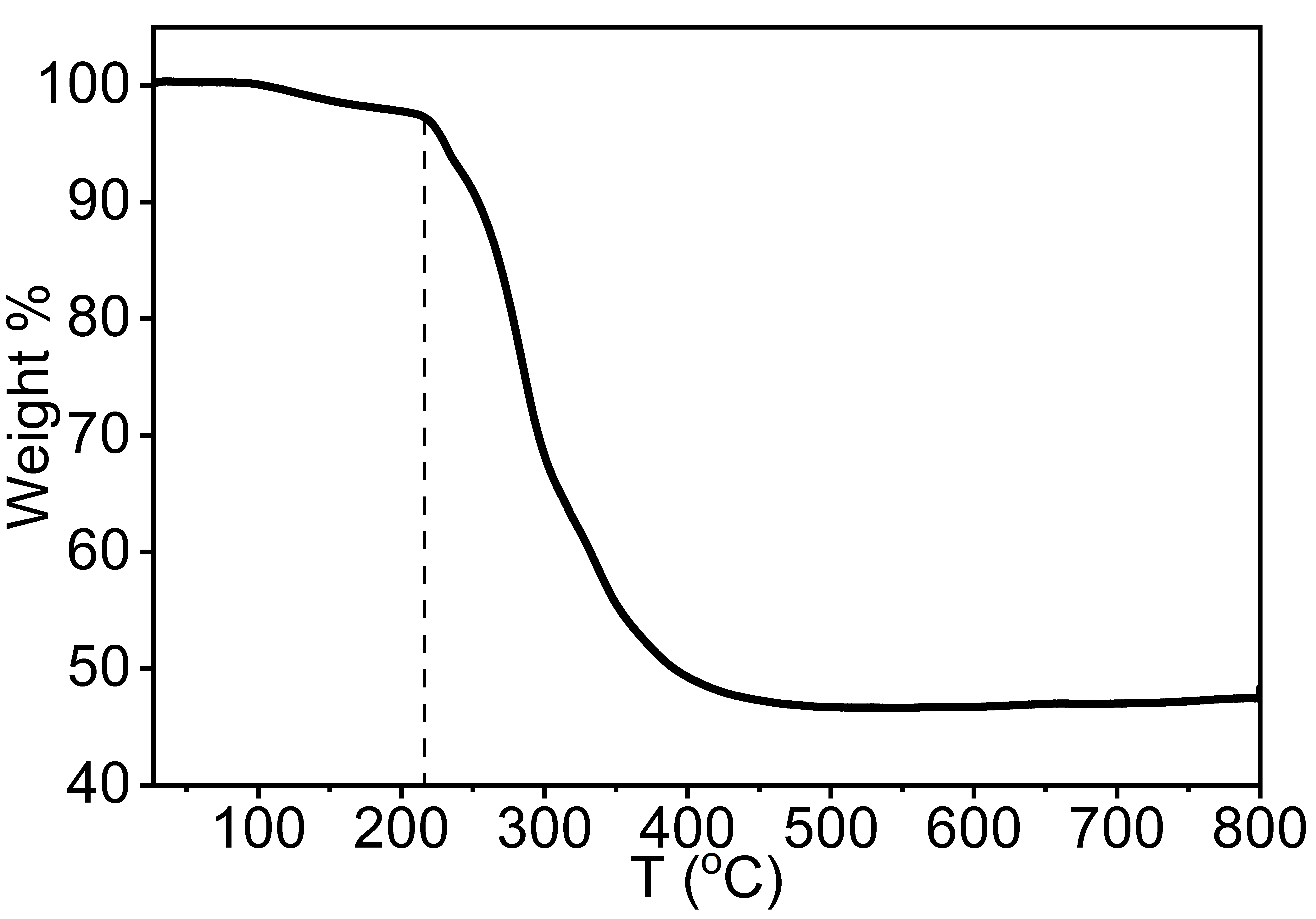


**Figure S15.** TGA of Ag_40_ nanocrystals.


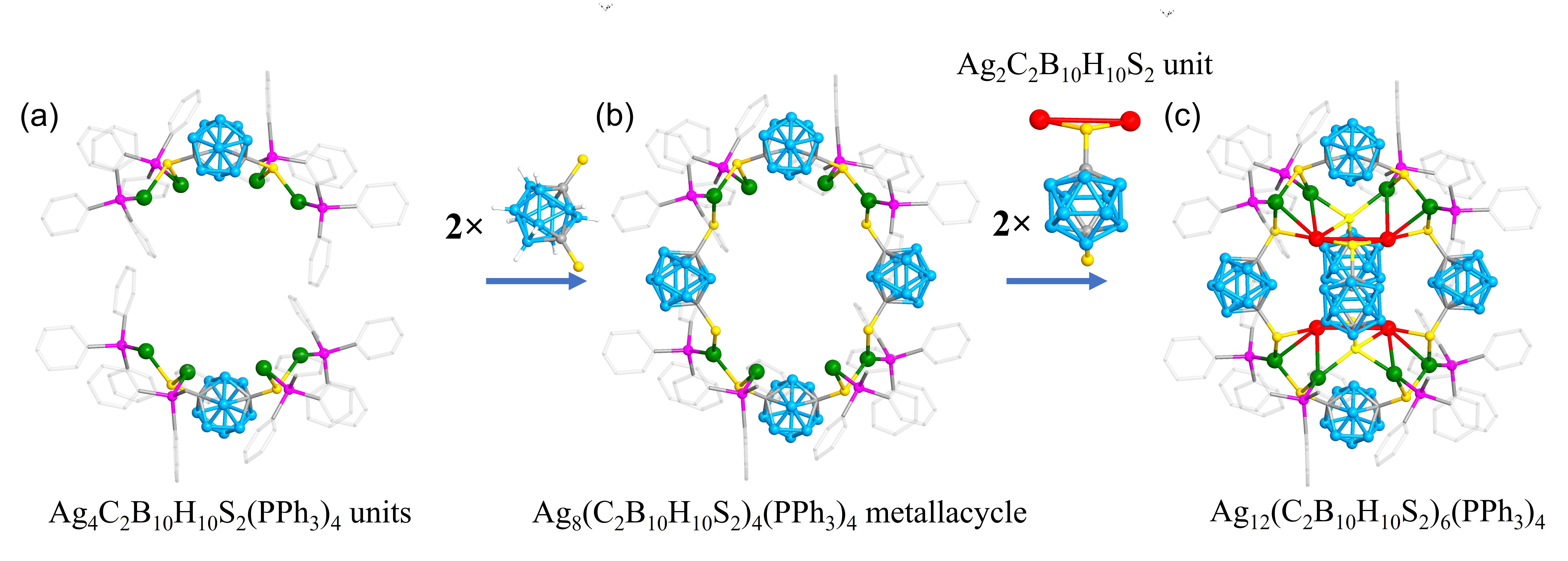


**Figure S16.** Anatomy of the **Ag_12_**. (a) Two Ag_4_C_2_B_10_H_10_S_2_(PPh_3_)_4_ units. (b) Ag_8_(C_2_B_10_H_10_S_2_)_4_(PPh_3_)_4_ metallacycle. (c) Molecule structure of Ag_12_(C_2_B_10_H_10_S_2_)_6_(PPh_3_)_4_. Color labels: red, blue, and green, Ag; yellow, S; purple, P; grey, C; cyan, B. All hydrogen atoms are omitted for clarity.


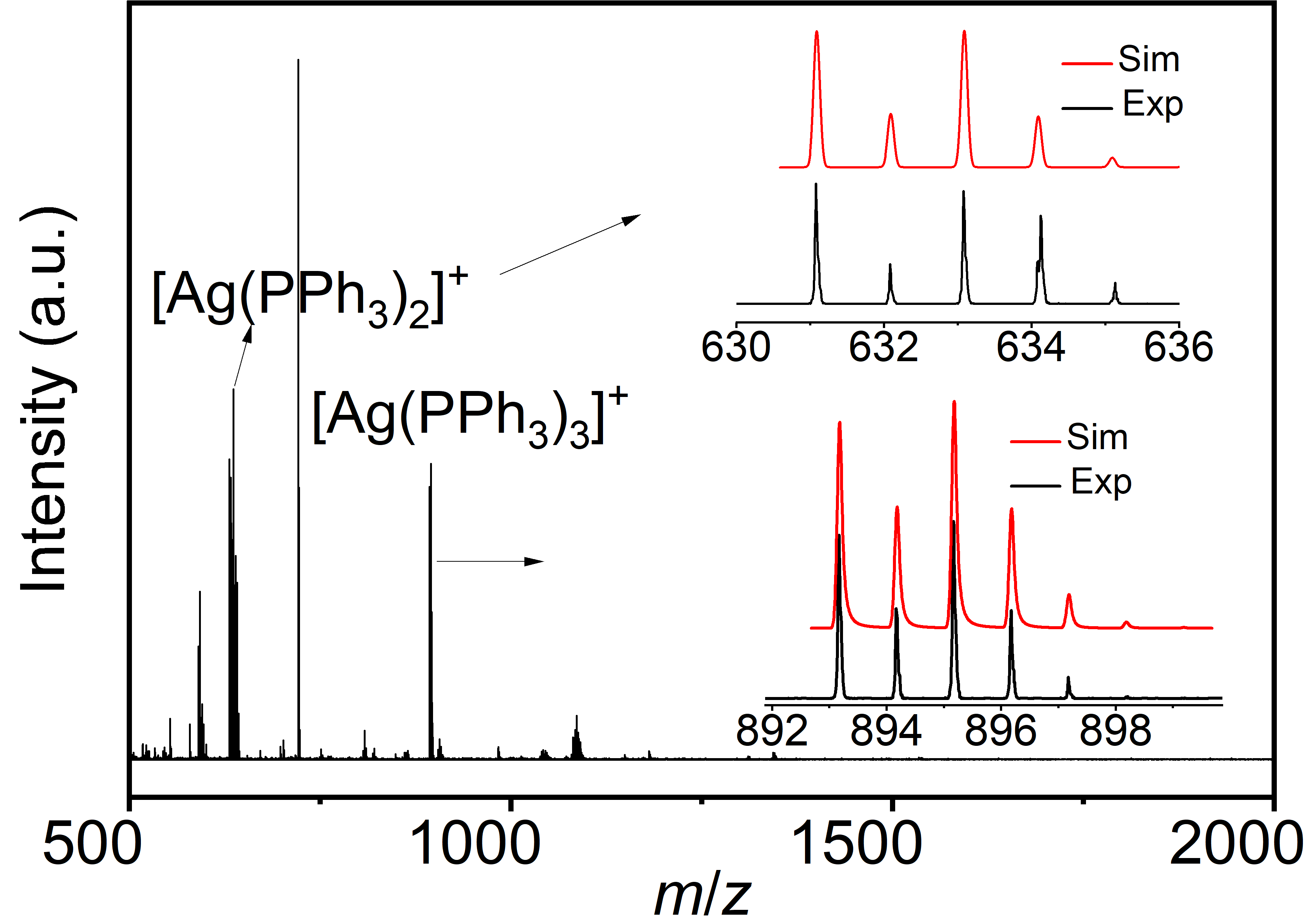


**Figure S17.** Positive-ion ESI-MS spectrum of [Ag_40_(C_2_B_10_H_10_S_2_)_12_(PPh_3_)_8_][Ag(PPh_3_)_4_]_2_. [Ag(PPh_3_)_4_]^+^ was not stable in the gas phase, and it fragmented into [Ag(PPh_3_)_3_]^+^ and [Ag(PPh_3_)_2_]^+^.^12^

^
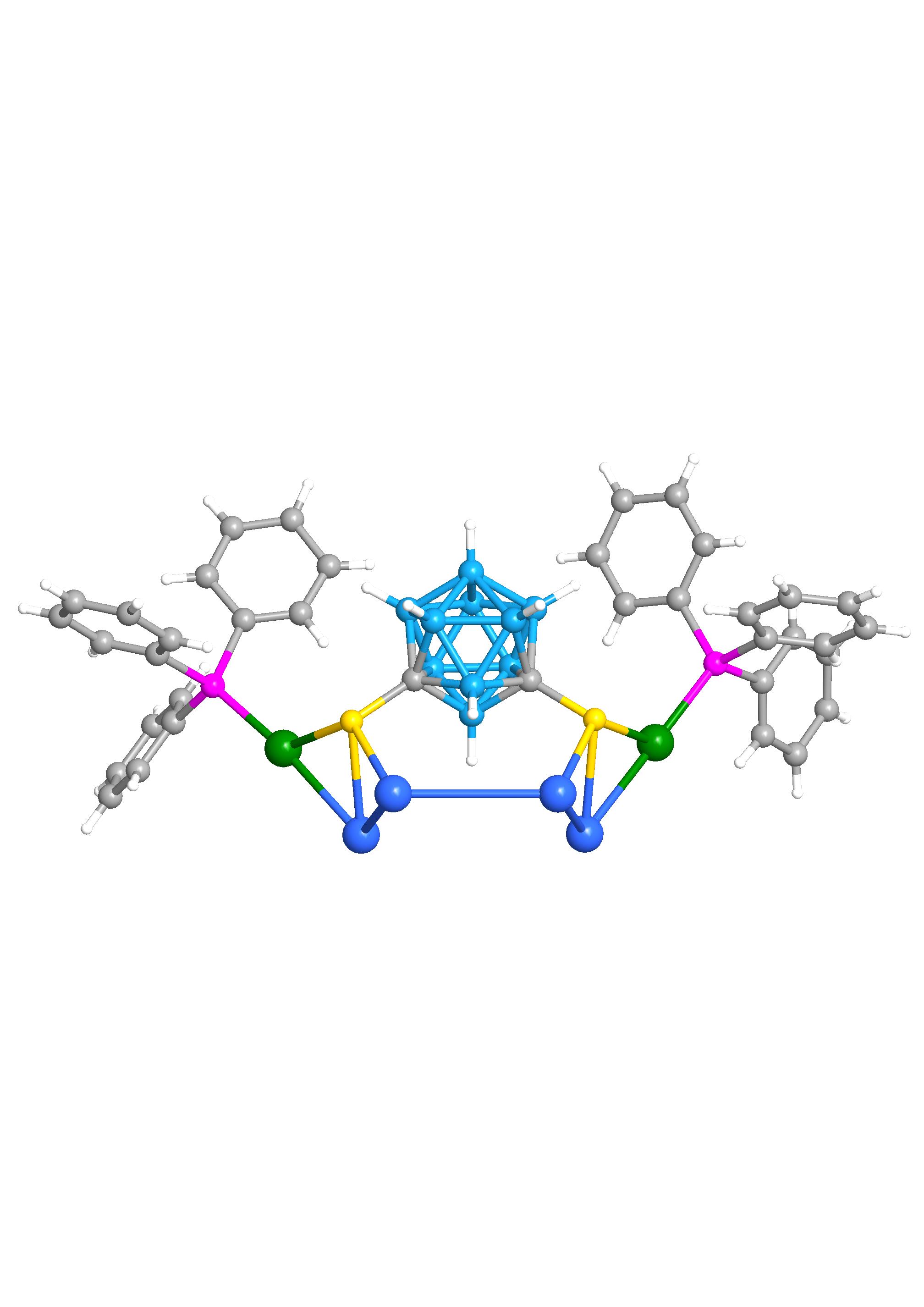
^

**Figure S18.** The coordination modes of S and P atoms. Color labels: blue, and green, Ag; yellow, S; purple, P; grey, C; cyan, B.


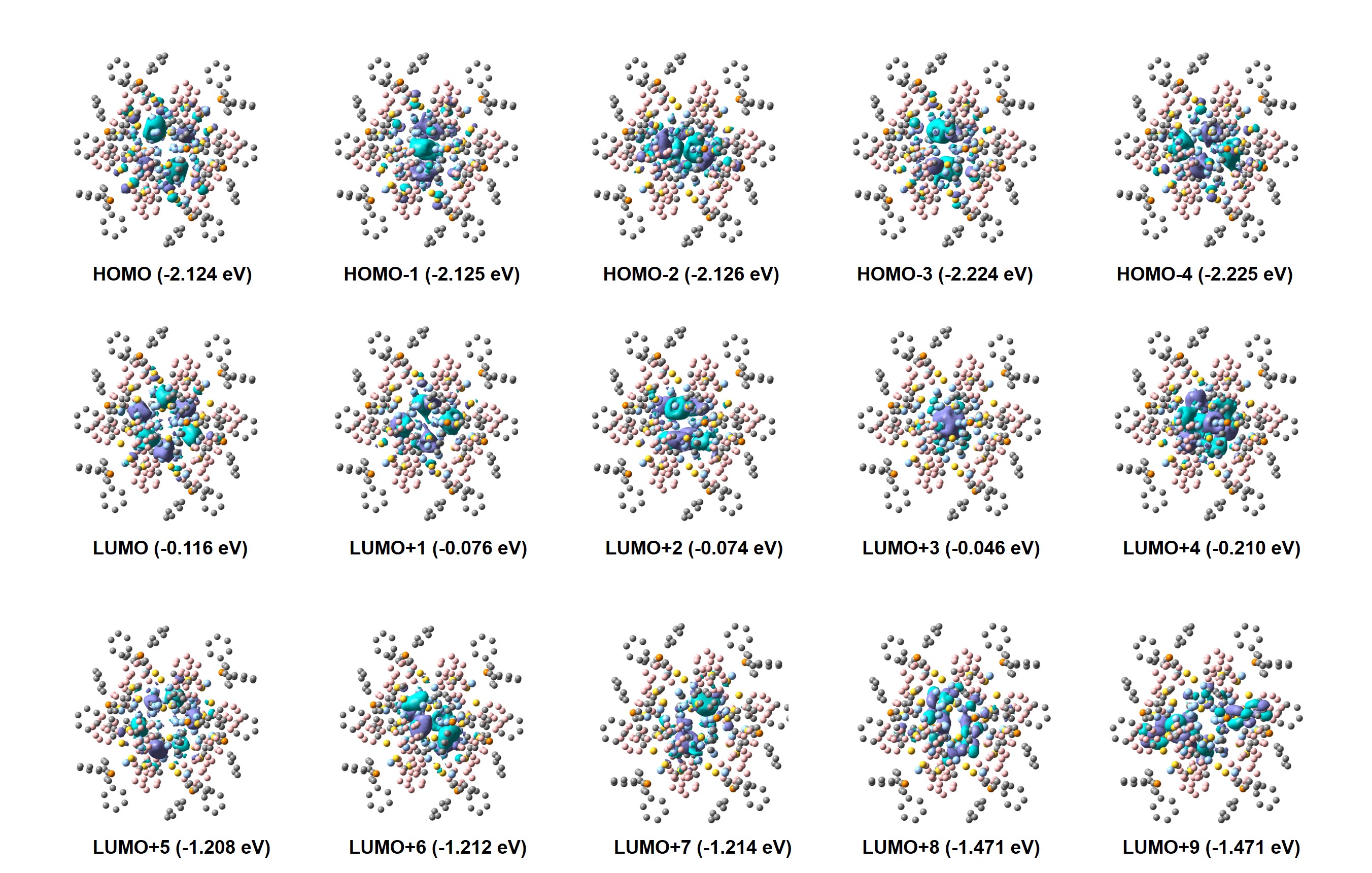


**Figure S19.** Selected molecular orbitals of the **Ag_40_** nanocluster. Color codes: light blue, Ag; yellow, S; orange, P; gray, C. For clarity, all H atoms are omitted.


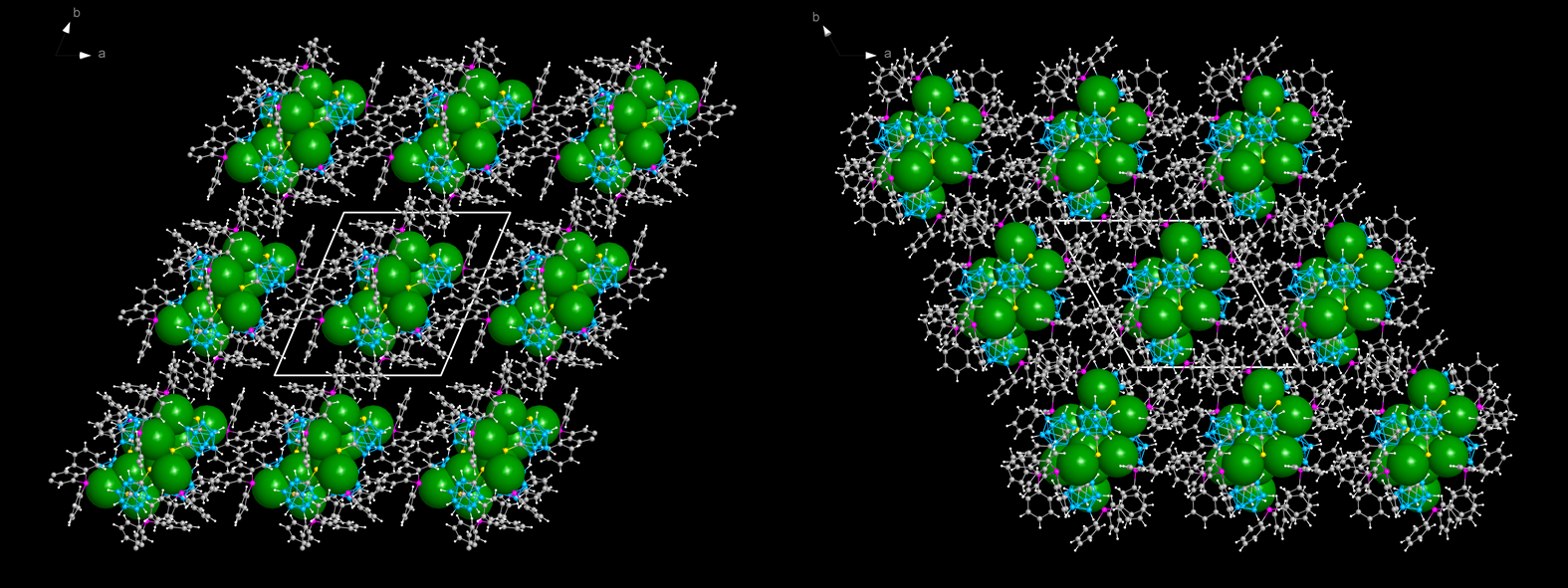


**Figure S20.** The packing of the Ag_12_ clusters in the lattice of **Ag_12_**.


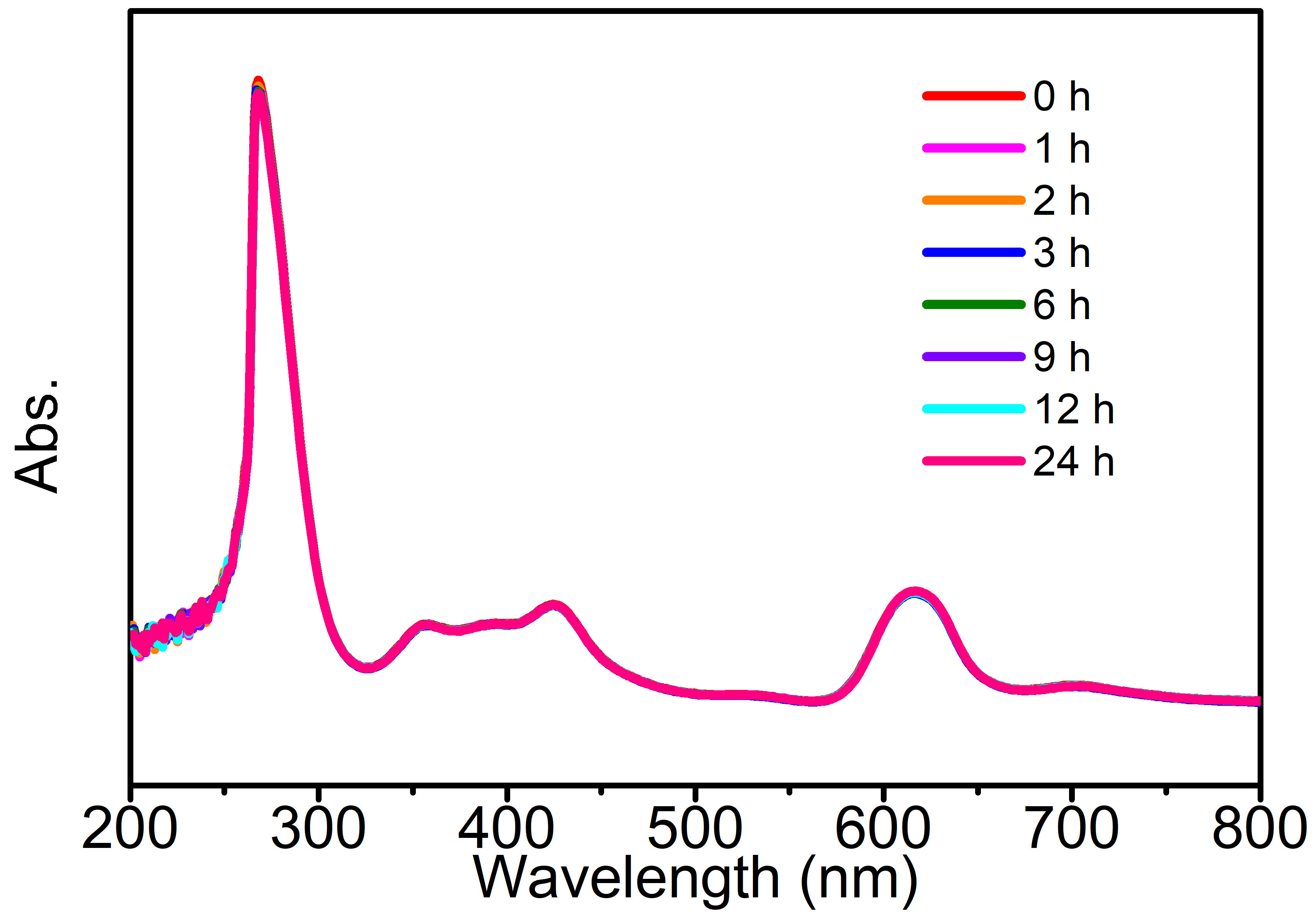


**Figure S21.** Time-dependent UV-vis absorption spectra of **Ag_40_** in the presence of small amount of PPh_3_. The increase of the absorption intensity at 267 nm was ascribed to the additional PPh_3_.


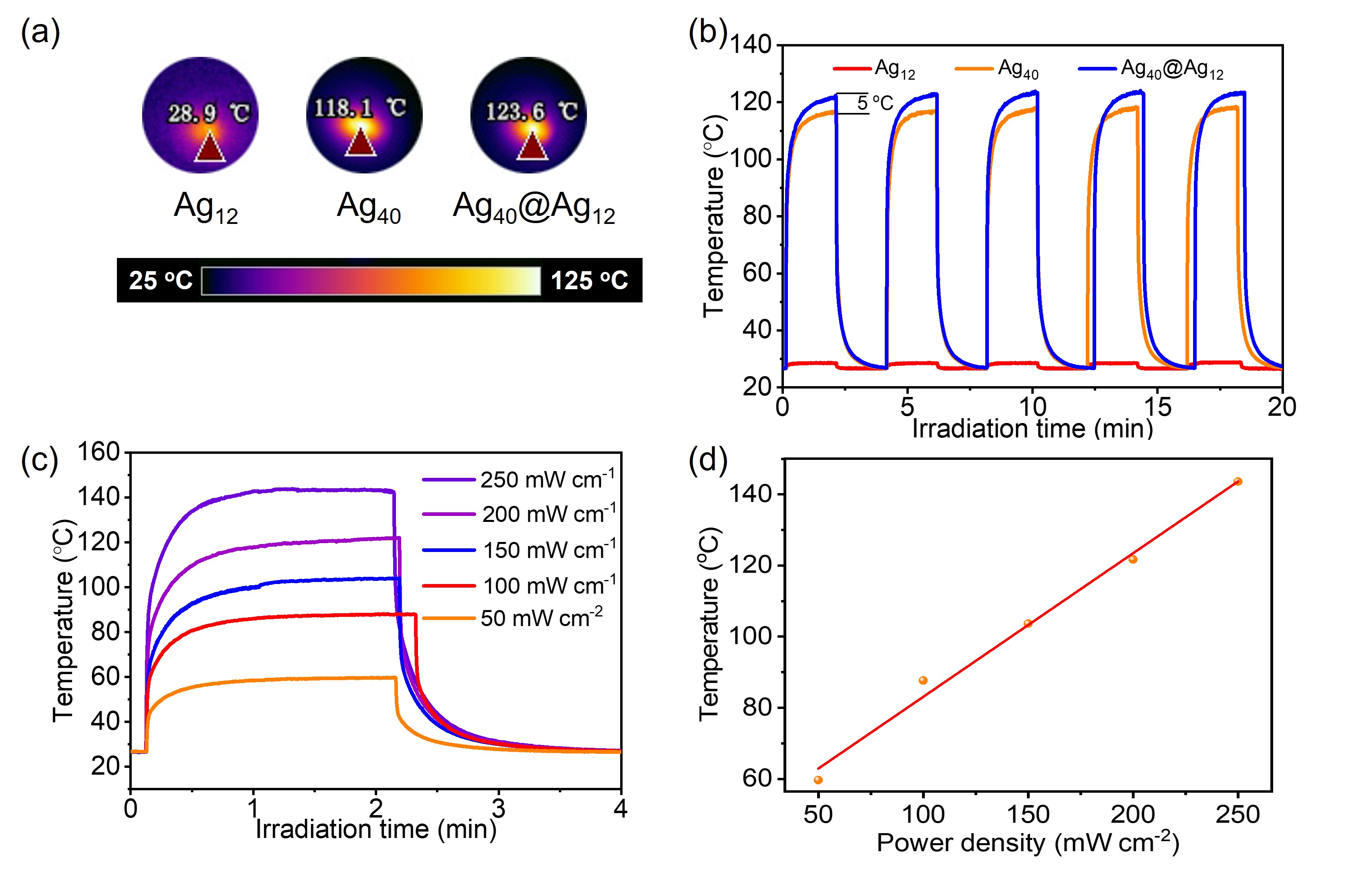


**Figure S22.** (a) Infrared thermal images under laser exposure (200 mW cm^-2^, 808 nm laser). (b) Photothermal heating and cooling cycles under laser exposure (200 mW cm^-2^, 808 nm laser). (c) Photothermal response of **Ag_40_@Ag_12_** under different power density laser exposure. (d) Plot of power density versus temperature of **Ag_40_@Ag_12_**.

**Table S1.** Crystal and structure determination data

| Identification code | Ag_12_ | Ag_40_ | Ag_40_@Ag_12_ |
| --- | --- | --- | --- |
| CCDC number | 2322648 | 2322649 | 2322650 |
| Empirical formula | C_168_H_208_Ag_12_B_60_N_4_O_4_P_8_S_12_ | C_120_H_150_Ag_51_B_60_P_12_S_30_ | C_162_H_194_Ag_12_B_60_N_2_O_2_P_8_S_12_ |
| Formula weight | 4922.89 | 9075.89 | 4776.70 |
| Temperature/K | 199.99(10) | 150.1(3) | 200.00(10) |
| Crystal system | triclinic | trigonal | triclinic |
| Space group | *P*-1 | *P*-3 | *P*-1 |
| a/Å | 17.0079(2) | 24.9371(4) | 16.9702(5) |
| b/Å | 18.4234(2) | 24.9371(4) | 18.3807(7) |
| c/Å | 20.4387(3) | 56.5447(9) | 20.4439(4) |
| α/° | 110 | 90 | 110 |
| β/° | 96 | 90 | 96 |
| γ/° | 114 | 120 | 114 |
| Volume/Å^3^ | 5182 | 30451 | 5167 |
| Z | 1 | 2 | 1 |
| ρ_calc_g/cm^3^ | 1.577 | 0.99 | 1.535 |
| μ/mm^‑1^ | -110.974 | -114.168 | -110.976 |
| F(000) | 2542 | 8454 | 2372 |
| Crystal size/mm^3^ | 0.02 × 0.02 × 0.01 | 0.1 × 0.1 × 0.05 | 0.04 × 0.04 × 0.01 |
| Radiation | Cu Kα (*λ* = 1.54184) | Cu Kα (*λ* = 1.54184) | Cu Kα (*λ* = 1.54184) |
| 2*θ* range for data collection/° | 5.75 to 147.668 | 4.38 to 130.17 | 5.746 to 150.382 |
| Index ranges | -20 ≤ *h* ≤ 21,  -21 ≤ *k* ≤ 22,  -25 ≤ *l* ≤ 25 | -29 ≤ *h* ≤ 19,  -26 ≤ *k* ≤ 27,  -40 ≤ *l* ≤ 66 | -21 ≤ *h* ≤ 20,  -20 ≤ *k* ≤ 22,  -24 ≤ *l* ≤ 25 |
| Reflections collected | 53528 | 96122 | 57573 |
| Independent reflections | 20237 [*R_int_* = 0.0924, *R_sigma_* = 0.0933] | 34324 [*R_int_* = 0.094, *R_sigma_* = 0.095] | 20313 [*R_int_* = 0.0756, *R_sigma_* = 0.0769] |
| Data/restraints/parameters | 20237/30/1211 | 34324/3838/1342 | 20313/52/1210 |
| Goodness-of-fit on F^2^ | 1.050 | 1.029 | 1.041 |
| Final R indexes [I>=2σ (I)] | *R_1_* = 0.0755,  *wR_2_* = 0.1979 | *R_1_* = 0.1240,  *wR_2_* = 0.3860 | *R_1_* = 0.0708,  *wR_2_* = 0.1914 |
| Final R indexes [all data] | *R_1_* = 0.0928,  *wR_2_* = 0.2057 | *R_1_* = 0.134,  *wR_2_* = 0.4034 | *R_1_* = 0.0916,  *wR_2_* = 0.2037 |
| Largest diff. peak/hole / e Å^-3^ | -32.31/-1.35 | 33.82/-3.57 | -32.9/-1.82 |

**References**

(1) Vinas, C.; Benakki, R.; Teixidor, F.; Casabo, J., Dimethoxyethane as a Solvent for the Synthesis of C-Monosubstituted *o*-Carborane Derivatives. *Inorg. Chem.* **1995**, *34*, 3844-3845.

(2) CrysAlisPro 2012, Agilent Technologies. Version 1.171.36.31.

(3) Sheldrick, G. M. *Acta Cryst. A* **2015**, *71*, 3-8.

(4) Dolomanov, O. V.; Bourhis, L. J.; Gildea, R. J.; Howard, J. A. K.; Puschmann, H. *J. Appl. Cryst.*

**2009**, *42*, 339-341.

(5) Spek, A. L. *Acta Cryst. C* **2015**, *71*, 9-18.

(6) Brandenburg, K. Diamond, **2010**.

(7) Bannwarth, C.; Caldeweyher, E.; Ehlert, S.; Hansen, A.; Pracht, P.; Seibert, J.; Spicher, S.; Grimme, S. *Comput. Mol. Sci.* **2020**, *11*, e1493.

(8) Bannwarth, C.; Ehlert, S.; Grimme, S. *J. Chem. Theory Comput.* **2019**, *15*, 1652−1671.

(9) Grimme, S.; Bannwarth, C.; Shushkov, P. *J. Chem. Theory Comput.* **2017**, *13*, 1989−2009.

(10) S. Grimme, J. Chem. Phys. **2013**, *138*, 244104.

(11) S. Grimme & C. Bannwarth, JCP **2016** *145*, 054103.

(12) Priola E., Bonometti E., Rabezzana R., Buscaino R., Michele R. Chierotti, Operti L., Diana E., *Inorg. Chem. Commun.*, **2016**, *70*, 35-40.
